# Tetanus Toxin Synthesis is Under the Control of A Complex Network of Regulatory Genes in *Clostridium tetani*

**DOI:** 10.3390/toxins12050328

**Published:** 2020-05-15

**Authors:** Diana Chapeton-Montes, Lucile Plourde, Cecile Deneve, Dominique Garnier, Fabien Barbirato, Vincent Colombié, Sandy Demay, Georges Haustant, Olivier Gorgette, Christine Schmitt, Catherine Thouvenot, Holger Brüggemann, Michel R. Popoff

**Affiliations:** 1 Bactéries anaérobies et Toxines, Institut Pasteur, 75724 Paris, France; dianajoanne@gmail.com (D.C.-M.); cecile.deneve-larrazet@u-psud.fr (C.D.); georges.haustant@pasteur.fr (G.H.); 2 Sanofi-Pasteur, 69280 Marcy l’Etoile, France; Lucile.Plourde@sanofi.com (L.P.); Dominique.Garnier@sanofi.com (D.G.); Fabien.Barbirato@sanofi.com (F.B.); Vincent.Colombie@sanofi.com (V.C.); Sandy.Demay@sanofi.com (S.D.); 3 Unité Technologie et Service Bioimagerie Ultrastructurale, Institut Pasteur, 75724 Paris, France; olivier.gorgette@pasteur.fr (O.G.); christine.schmitt@pasteur.fr (C.S.); catherine.thouvenot@pasteur.fr (C.T.); 4 Department of Biomedicine, Aarhus University, 8000 Aarhus, Denmark; brueggemann@biomed.au.dk

**Keywords:** *Clostridium tetani*, *Clostridium botulinum*, tetanus toxin, two-component system, gene transcription

## Abstract

*Clostridium tetani* produces a potent neurotoxin, the tetanus toxin (TeNT), which is responsible for an often-fatal neurological disease (tetanus) characterized by spastic paralysis. Prevention is efficiently acquired by vaccination with the TeNT toxoid, which is obtained by *C.* *tetani* fermentation and subsequent purification and chemical inactivation. *C.* *tetani* synthesizes TeNT in a regulated manner. Indeed, the TeNT gene (*tent*) is mainly expressed in the late exponential and early stationary growth phases. The gene *tetR (*tetanus regulatory gene*),* located immediately upstream of *tent*, encodes an alternative sigma factor which was previously identified as a positive regulator of *tent*. In addition, the genome of *C.* *tetani* encodes more than 127 putative regulators, including 30 two-component systems (TCSs). Here, we investigated the impact of 12 regulators on TeNT synthesis which were selected based on their homology with related regulatory elements involved in toxin production in other clostridial species. Among nine TCSs tested, three of them impact TeNT production, including two positive regulators that indirectly stimulate *tent* and *tetR* transcription. One negative regulator was identified that interacts with both *tent* and *tetR* promoters. Two other TCSs showed a moderate effect: one binds to the *tent* promoter and weakly increases the extracellular TeNT level, and another one has a weak inverse effect. In addition, CodY (control of dciA (decoyinine induced operon) Y) but not Spo0A (sporulation stage 0) or the DNA repair protein Mfd (mutation frequency decline) positively controls TeNT synthesis by interacting with the *tent* promoter. Moreover, we found that inorganic phosphate and carbonate are among the environmental factors that control TeNT production. Our data show that TeNT synthesis is under the control of a complex network of regulators that are largely distinct from those involved in the control of toxin production in *Clostridium botulinum* or *Clostridium difficile*.

## 1. Introduction

*Clostridium tetani* is an environmental Gram-positive, spore-forming and anaerobic rod-shaped bacterium which synthesizes a potent neurotoxin, the tetanus toxin (TeNT) [1]. *C. tetani* spores can enter an organism through an open wound. Spores germinate and *C. tetani* grows in anaerobic conditions in necrotic tissues. TeNT is synthesized at the end of the exponential growth phase and is released in the surrounding tissues. TeNT specifically recognizes nidogens at the neuromuscular junctions [2], and enters motorneurons. TeNT is retrogradely transported to the central nervous system. Then, TeNT enters inhibitory interneurons and blocks the release of neurotransmitters (glycine, GABA (gamma-aminobutyric acid)) upon proteolytic cleavage of the SNARE (soluble N-ethylmaleimide-sensitive factor attachment protein receptor) protein VAMP2 (vesicle associated membrane protein) [3,4].

Vaccination based on the TeNT toxoid is a very efficient prevention measure against tetanus. It is noteworthy that in addition to the use as a single-antigen vaccine for specific prevention of tetanus, the TeNT toxoid is combined with other vaccine antigens for protection against other infectious diseases [5]. Industrial TeNT production is obtained by fermentation of *C. tetani* clinical isolates. TeNT is then extracted from culture supernatants and inactivated with formalin. *C. tetani* fermentation is a critical and complex step in vaccine production as it is performed in a rich growth medium under a strictly controlled environment. The essential medium components such as amino acids that result in a high TeNT yield are partially identified. Thus, the toxin yield varies with different media and charges [6].

The TeNT gene (*tent*) is located on a large plasmid in *C. tetani* [7,8]. The first complete genome sequence of a toxigenic *C. tetani* strain (E88) was determined in 2003 [9]. The genome contains a chromosome of approximately 2.8 Mb and a TeNT-encoding plasmid of 74 kb. The genomes of additional *C. tetani* strains have been sequenced and showed that the plasmid-encoded *tent* is highly conserved [10,11,12]. A conserved gene (*tetR,* tetanus toxin regulatory gene) just upstream of *tent* encodes for an alternative sigma factor which positively regulates the transcription of *tent* [13]. TetR is homologous to BotR (botulinum toxin regulator) which controls the synthesis of the botulinum neurotoxin (BoNT) in *Clostridium botulinum* A, B, C and D [14]. TetR and BotR belong with *Clostridium difficile* TcdR (or TxeR) (*Clostridium difficile* toxin regulator) and *Clostridium perfringens* UviA (UV-inducible gene A) to a sub-group of the sigma 70 family of RNA polymerase sigma factors which control clostridial toxin gene syntheses [15,16]. However, the regulatory network governing TeNT synthesis is still poorly understood. A better knowledge of the regulatory mechanisms of TeNT synthesis in *C. tetani* and determination of the environmental factors controlling this regulation are required for improving toxin production.

To successfully respond to changes in different environmental conditions and to regulate virulence, many bacteria use a complex regulatory network involving diverse types of molecules, including RNA, DNA, proteins and metabolites. Among these are global regulators like two-component systems (TCS) and CodY to sense the relevant environmental signals [17,18]. TCSs are ubiquitous among bacteria. They consist of a membrane-bound or cytosolic sensor histidine kinase (SHK) that senses a stimulus and its cytoplasmic cognate response regulator (RR) that mediates the cellular response. Following a specific stimulus, the SHK autophosphorylates at a conserved histidine residue. The phosphoryl group is then transferred from the histidine to a conserved aspartate residue in the RR, which upon phosphorylation is able to control the expression of its target genes [18].

Depending on the availability of nutrients, bacteria have to adjust their gene expression. The global regulator CodY has been shown to be an important regulatory link between metabolism and virulence factor synthesis in many low G + C Gram-positive bacteria. CodY displays enhanced affinity for its DNA target when bound to GTP and/or branched-chain amino acids [19]. In *C. botulinum* ATCC 3502, CodY has been shown to positively regulate *bont* expression, and to bind to the promoter of the *ntnh-bontA* operon in a GTP-dependent manner [20]. In addition, the sporulation regulator Spo0A has been found to positively regulate BoNT synthesis in *C. botulinum* type E, which is part of the group II of *C. botulinum* and lacks BotR. Spo0A binds to the promoter of the *bont/E* operon and enhances its transcription [21]. Spo0A is expressed in the exponential growth phase of *C. botulinum* and initiates the cascade of alternative sigma factors involved in sporulation [22]. However, Spo0A targets several hundred other genes and has pleiotropic effects [23]. Moreover, the mutation frequency decline (*mfd*) gene, which encodes a transcription-coupled repair factor, also shows pleiotropic effects: it increases the expression of both Toxin A and Toxin B-encoding genes in *C. difficile* [24]. These genes *codY, spo0A* and *mfd* are conserved in the *C. tetani* genome.

Genomic analysis of the *C. tetani* strain E88 reveals the presence of numerous putative regulatory genes, some of them being homologous to regulatory genes in other clostridia or bacteria. The aim of this work was to determine the role of 12 putative regulatory systems in TeNT synthesis, nine two-component systems as well as the global regulators CodY, Spo0A and Mfd.

## 2. Results

### 2.1. Genomic Analysis of Regulatory Genes in C. tetani 

Genome sequencing of strain E88 showed that the large *tent*-containing plasmid also harbors regulatory elements: *tetR* located immediately upstream of *tent*, a two-component system (TCS) (CTC_RS13805/CTC_RS13810, previously named *ctp21/ctp22*) homologous to systems in other Gram-positive bacteria including *C. botulinum* and *Bacillus* sp., and three additional putative sigma factors [9]. In addition, refined genomic analysis showed that the chromosome of strain E88 possesses 30 TCSs based on the conserved motifs of histidine kinase as part of the SHK and a receiver domain Rec as part of the adjacent RR. Most of the 30 TCSs belong to the OmpR (outer membrane protein regulator) family (19 TCSs), two to (lytic gene regulator/alginate biosynthesis regulator) LytR/AlgR, two to (nitrate reductase regulator) NarL, two to (factor for inversion stimulation) Fis, one to (L-arabinose gene regulator) AraC, one to (purine catabolic gene regulator) PucR, one to LysR (lysine synthesis regulator) and one to XRE ((prophage *Bacillus subtilis*) PBSX repressor) families (Table 1). All 30 TCSs except one share homology with putative TCSs in other clostridia species. Among them, 19 TCSs are homologous to related genes in *C. botulinum* strain Hall (identity level ≥ 45%) (Table 1). It was previously shown that five TCSs in strain Hall positively regulate botulinum neurotoxin (BoNT) synthesis [25]. Two of them are homologous to *C. tetani* TCSs, i.e., CLC0661/CLC0663 from *C. botulinum* with 65% protein identity to CTC_RS02080/CTC_RS02085 of *C. tetani* and CLC0410/CLC0411 shows 68% protein identity to CTC_RS10030/CTC_RS10035 (Table 1). Another study identified one TCS (CB00786/CB00787) in *C. botulinum* strain ATCC3502, that negatively regulates *bont* gene expression [26]. This TCS shares significant identity with a *C. tetani* TCS (CTC_RS07310/CTC_RS07315).

The genome of *C. tetani* strain CN655 [10] shares the same TCS genes as strain E88. Since we have already characterized the *tetR* in strain CN655, a strain which is more easily transformable compared to other *C. tetani* strains under the applied conditions, the strain CN655 was selected for further genetic investigations including the analysis of nine TCS genes, *mfd*, *spo0A* and *codY.*

Res, respiration; Bac, bacitracin; Vir, virulence; Spa, subtilin gene; Van, vancomycin; Gtc, gramicidin transcription; Arc, aerobic respiration control; Pho, phosphate; Feu, ferric uptake; Dcu, dicarboxylate uptake; Sin, sporulation inhibition; Eut, ethanolamine utilization; Ato, acetoacetyl-coenzyme A transferase.

### 2.2. Construction of TCS Anti-Sense Strains 

Plasmids that were able to generate anti-sense mRNA from nine TCS genes, as well as from *mfd*, *spo0A* and *codY*, were constructed in analogy to the construction of pMRP306 which has previously been used in the investigation of *tetR* in *C. tetani* and regulatory genes in *C. botulinum* [13,14]. DNA segments for anti-sense mRNA production were designed, located in the RR gene of three TCSs (CTC_RS04785, CTC_RS10155, and CTC_RS07315) and in the SHK gene in six other TCSs (CTC_RS13810, CTC_RS02085, CTC_RS04705, CTC_RS06180, CTC_RS05750 and CTC_RS10030) (Table 2). 

Although the growth kinetics of CN655 harboring the empty vector pAT18 (CN655/pAT18) was slightly lower than that of the wt CN655 during the exponential growth phase and beginning of the stationary phase, the total production of TeNT was slightly higher (Supplementary Materials Figure S1). Both CN655/pAT18 and CN655 wt showed similar growth and toxin production from 72 h to 144 h (Supplementary Materials Figure S1). No difference in bacterial size and microscopic morphology was observed between CN655 wt and CN655/pAT18.

**Figure 1:**
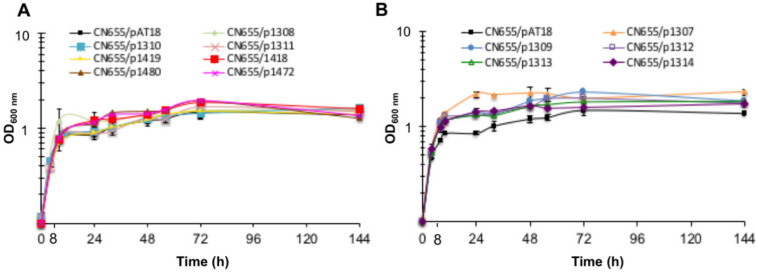
Growth kinetics of *C. tetani* strain CN655/pAT18 (empty vector) and CN655 antisense strains. (**A**) CN655/p1308, CN655/p1310, CN655/p1311, CN655/p1419, CN655/p1418, CN655/1480 and CN655/p1472 showed a similar kinetics compared to CN655/pAT18 strain. (**B**) CN655/p1307, CN655/p1309, CN655/p1312, CN655/p1313 and CN655/p1314 displayed a more abundant growth than CN655/pAT18 in the early stationary phase (12–48 h). Data are mean values ± SEM of at least three independent cultures.

Seven CN655 anti-sense strains targeting four TCSs (p1308 targeting CTC_RS02085, p1310 targeting CTC_RS04785, p1311 targeting CTC_RS05750, p1419 targeting CTC_RS07315), and three regulators (p1480 targeting *mfd*, p1472 targeting *spo0A,* and p1418 targeting *codY*), showed similar growth kinetics compared to the control strain CN655/pAT18 (Figure 1). However, growth of strains CN655/p1307 targeting CTC_RS13810, CN655/p1309 targeting CTC_RS04705, CN655/p1312 targeting CTC_RS06180, CN655/p1313 targeting CTC_RS10030, and CN655/p1314 targeting CTC_RS10155 was more abundant than the control strain CN655/pAT18 in the early stationary phase (12–48 h), but the five anti-sense strains reached a similar biomass as monitored by OD_600nm_ than that of the other anti-sense strains at 72–144 h (Figure 1).

### 2.3. TeNT Synthesis is Altered in Five TCS Anti-Sense Strains and in the codY Anti-Sense Strain

**Figure 2:**
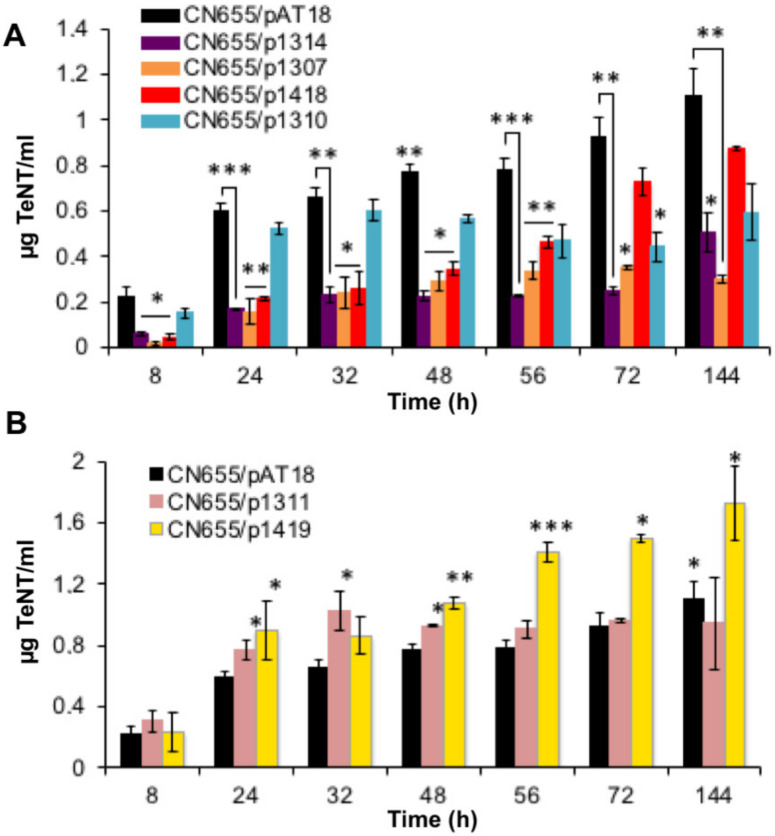
Extracellular tetanus toxin (TeNT) produced by *C. tetani* CN655/pAT18 (empty vector) and CN655 antisense strains. (**A**) Extracellular toxin was reduced in the culture supernatant of CN655/p1307, CN655/p1310, CN655/p1314 and CN655/p1418. (**B**) CN655/p1311 and CN655/p1419 showed elevated extracellular toxin kinetics compared to control strain CN655/pAT18. Statistical significance of differences between control and anti-sense strains is indicated with *p*-values (*, *p* < 0.05; **, *p* < 0.01; ***, *p* < 0.001). Data are mean values ± SEM of at least three independent cultures.

Extracellular TeNT concentrations as monitored by enzyme-linked immunosorbent assay (ELISA) in the culture supernatant were reduced in three CN655 anti-sense strains (CN655/p1307, CN655/p1314 and CN655/p1310) as well as in the *codY* anti-sense strain (CN655/p1418) compared to the control strain CN655/pAT18 (Figure 2A). CN655/p1307, which targets the TCS located on the large *tent*-containing *C. tetani* plasmid, and CN655/p1314 targeting the chromosomal CTC_RS10155 TCS, showed a drastic decrease (65% to 75%) in the levels of secreted TeNT. In contrast, CN655/p1310 which targets CTC_RS04785 exhibited a moderate decrease (about 25%) in secreted TeNT. The *codY* anti-sense strain (CN655/p1418) showed a significantly lower (50% to 65%) TeNT level in the culture supernatant within the first 56 h of culture and a less pronounced effect (20% to 25%) in the late growth phase (Figure 2A). The anti-sense strains CN655/p1308, CN655/p1309, CN655/p1312, CN655/p1313, CN655/p1480 and CN655/p1472 showed no significant difference in the production of extracellular TeNT compared to CN655/pAT18 (Supplementary Figure S1).

**Figure 3:**
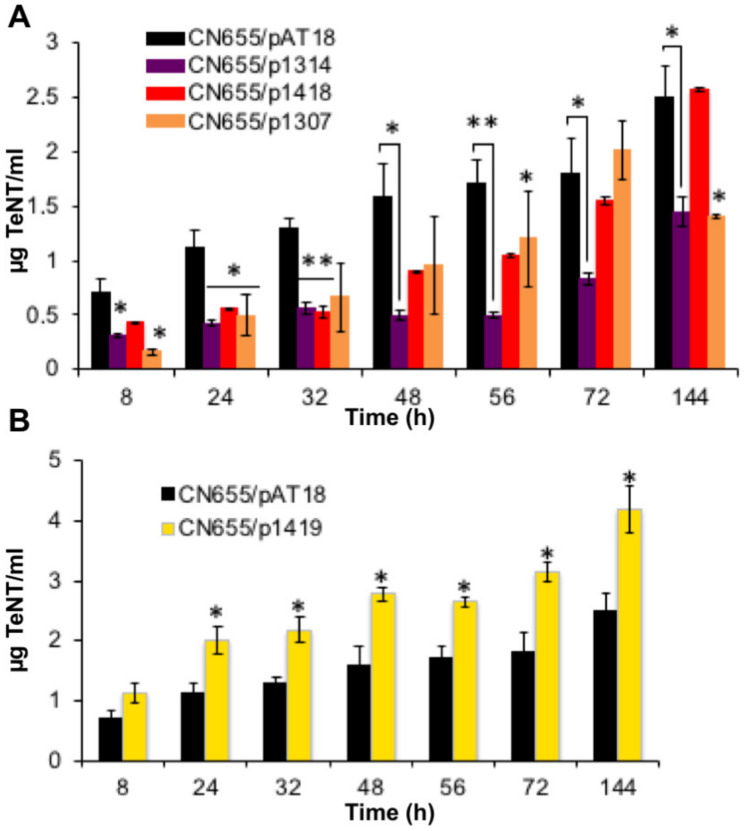
Total tetanus toxin (TeNT) produced by *C. tetani* CN655/pAT18 (empty vector) and CN655 antisense strains. (**A**) Total tetanus toxin production was reduced in CN655/p1307, CN655/p1314 and CN655/p1418. (**B**) CN655/p1419 showed elevated total toxin kinetics compared to control strain CN655/pAT18. Three independent experiments have been done. Statistical significance of differences between control strain and anti-sense strains is indicated with *p*-values (*, *p* < 0.05; **, *p* < 0.01).

Total amounts of produced TeNT (i.e., TeNT in the culture supernatant and intracellular TeNT at the end of culture growth) were significantly decreased in CN655/p1307, CN655/p1314 and in CN655/p1418, whereas no significant difference was observed in CN655/p1310 (Figure 3A).

In contrast, the extracellular and total TeNT concentrations were significantly increased in CN655/p1419 targeting CTC_RS07315 (Figure 2B and Figure 3B). The anti-sense strain CN655/p1311 targeting CTC_RS05750 showed an increase in extracellular TeNT within the first 48 h of culture, but the total TeNT amount at the end of culture was not significantly different from the control strain (Figure 2, Supplementary Materials Figure S2).

The total production of TeNT was not significantly different from that of the control strain CN655/pAT18 in CN655/p1308, CN655/p1309, CN655/p1312, CN655/p1313, CN655/p1480 and CN655/p1472 (Supplementary Materials Figure S3).

### 2.4. Five TCSs and codY Control Tent and/or Tetr at the Transcriptional Level

**Figure 4:**
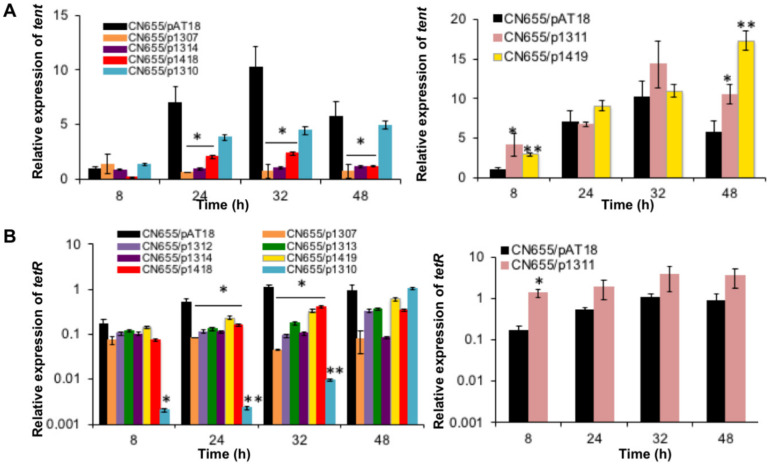
Expression of (**A**) *tent* and (**B**) *tetR* in CN655/pAT 18 and CN655 antisense strains. (**A**) The expression of *tent* was repressed in CN655/p1307, CN655/p1310, CN655/p1314 and CN655/p1418 compared to the control strain CN655/pAT18. For strains CN655/p1311 and CN655/p1419, an increased *tent* expression was observed. (**B**) The expression of *tetR* was repressed in CN655/p1307, CN655/p1310, CN655/p1312, CN655/p1313, CN655/p1314, CN655/p1419 and CN655/p1418. Strain CN655/p1311 showed an increase in *tetR* expression compared to the control strain CN655/pAT18. Target gene expression was normalized to *rpoB* and *gyrA*. Three independent experiments have been done. Statistical significance of differences between control and the anti-sense strains is indicated with *p*-values (*, *p* < 0.05; **, *p* < 0.01).

The transcriptional levels of *tent* and *tetR* were monitored by qRT-PCR at 8, 24, 32 and 48 h of culture, which corresponded to the exponential growth phase (8 h) and early stationary growth phase (12–48 h) (Figure 1). The limit of these analyses is based on the consideration that the copy numbers of the recombinant plasmids are similar in the recombinant strains. Among the TCSs investigated by RNA anti-sense, CN655/p1307 and CN655/p1314 showed a significant reduction of *tent* and *tetR* transcription during the 24–48 h growth phase and only a moderate effect at the 8 h exponential growth phase (Figure 4). A marked reduced transcription of *tetR* was observed in CN655/p1310 at the 8 h exponential and 24–32 h early stationary growth phase, whereas *tent* transcription was not significantly reduced (Figure 4). In contrast, the anti-sense strain CN655/p1311 showed an increase in *tent* and *tetR* transcription at the 8 h exponential growth phase and to a lower extent within the 24–48 h stationary growth phase (Figure 4). The anti-sense strain CN655/p1419 also exhibited an increased *tent* transcription level at 8 h exponential growth phase and at 48 h of culture, but a decreased transcription of *tetR* within the 24–32 h early stationary growth phase, albeit to a lower extent than in CN655/p1307 and CN655/p1314 (Figure 4). This suggests that CTC_ RS07315 (targeted in CN655/p1419) controls *tent* transcription independently of *tetR.*

The transcriptional levels of *tent* and *tetR* were not significantly different in CN655/p1308, and CN655/p1309 compared to the control strain CN655/pAT18 (Supplementary Materials Figure S4). Despite a slightly decreased transcription of *tetR* in CN655/p1312 and CN655/p1313 during the 24–32 h early stationary growth phase (Figure 4), no significant alteration of *tent* transcription was observed in these strains (Supplementary Materials Figure S3). 

The anti-sense strain in which *codY* was targeted (CN655/p1418)*,* showed a decrease in *tent* transcription within the 8–48 h culture and a moderate decrease in *tetR* transcription (Figure 4). 

### 2.5. The CTC_RS07315 and CTC_RS04785 Response Regulators as well as Cody Bind to the Tent Promoter

**Figure 5:**
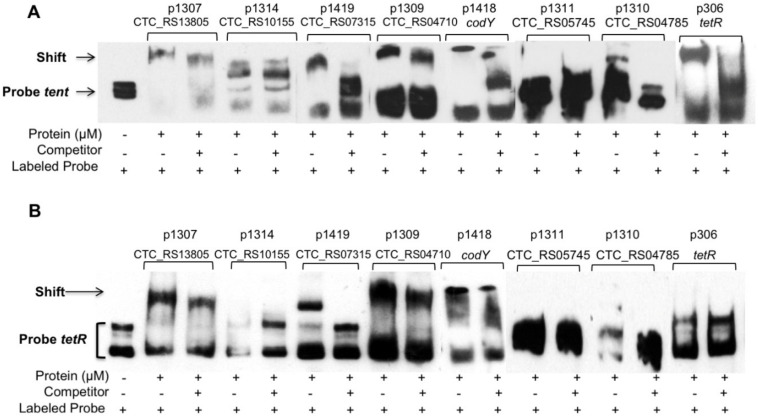
Electrophoretic mobility shift assay (EMSA) showing regulatory protein binding to *tent* (**A**) and *tetR* (**B**) promoters. Biotin-labeled DNA probes corresponding to the promoter regions of *tent* (P*_tent_*) and *tetR* (P*_tetR_*) were incubated with 5 μM of the recombinant proteins CTC_RS13805, CTC_RS10155, CTC_RS07315, CTC_RS04710, CTC_RS05745, CTC_RS04785, CodY and TetR. The specific binding of recombinant proteins to promoter probes resulted in an observable mobility shift when compared to the P*_tent_* and P*_tetR_* alone. Competition assays were performed with a 300-fold excess of unlabeled probe. Specificity of binding to P*_tent_* was confirmed for CTC_RS07315, CTC_RS04785, CodY and TetR. CTC_RS07315 was the only protein showing specific binding to P*_tetR_*. Addition of recombinant proteins, unlabeled promoter probe as cold competitor and labeled promoter probes are indicated. Representative experiments out of three are shown.

To identify the mode of action of the positive and negative regulators of *tent* gene transcription, we investigated their possible direct interaction with *tent* and/or *tetR* genes by testing the binding of the regulators to the promoters of *tent* and *tetR*. As shown in Figure 5, the CTC_ RS07315 (targeted in CN655/p1419) RR specifically bound to the promoters of both *tent* and *tetR* (P*_tent_,* P*_tetR_*). The RR CTC_RS04785 (targeted in CN655/p1310) caused a shift in the mobility of P*_tent_* but not in P*_tetR_*, despite the significant decrease in *tetR* transcription. In contrast, no specific binding to P*_tent_* or P*_tetR_* was observed with the RRs of CTC_RS10155, CTC_RS13805 and CTC_RS05745, albeit they influenced the TeNT synthesis and transcription of *tent.* As expected, no electrophoretic shift was observed with SHK such as CTC_RS04710. In addition, CodY bound to P*_tent_* but not to P*_tetR_.* As a positive control, TetR, which has been found to positively regulate *tent,* was used [13].

### 2.6. Inorganic Phosphate (P_i_) Influences TeNT Production

**Figure 6:**
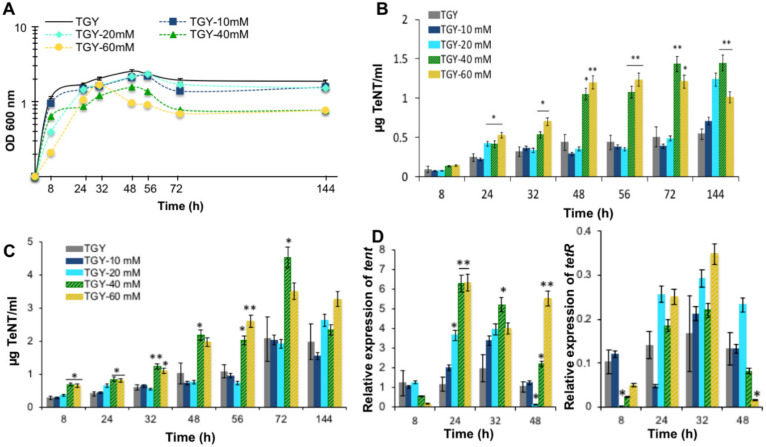
Effect of inorganic phosphate on tetanus toxin (TeNT) production, and *tent/tetR* expression. (**A**) Growth kinetics of CN655 in TGY supplemented with various concentrations of inorganic phosphate. (**B**) Extracellular TeNT levels. (**C**) Total TeNT levels. (**D**) Expression of *tent* and (**E**) *tetR*. Data are mean values ± SEM of at least three independent cultures. *, *p* < 0.05; **, *p* < 0.01.

Since two TCSs (CTC_RS05745/CTC_RS05750 and CTC_RS10030/CTC_RS10035) encoded in the *C. tetani* genome are putatively involved in the control of phosphate uptake and/or metabolism, as judged from their homologies with previously characterized TCSs, we checked the effect of inorganic phosphate (P_i_) in the culture medium on TeNT synthesis. Therefore, TGY (trpticase/glucose/yeast extract) culture medium containing less than 2 mM P_i_ was supplemented with 10 to 60 mM P_i_. *C. tetani* growth was similar in TGY supplemented with 10 and 20 mM P_i_ than that in the TGY control medium, but was slightly decreased when supplemented with 40 and 60 mM P_i_ (Figure 6). Extracellular and total TeNT production was increased in TGY containing 40 or 60 mM P_i_, whereas no significant difference in TeNT production was observed in TGY supplemented with 10 or 20 mM P_i_. The highest level of TeNT production occurred when *C. tetani* was grown in TGY medium with 40 mM P_i_ (at least two-fold more extracellular TeNT compared to TGY control medium) (Figure 6).

Expression of *tent* was increased in TGY medium containing 10 or 20 mM Pi, and to a higher extent when supplemented with 40 or 60 mM Pi (Figure 6D). The kinetics of increased *tent* expression in TGY supplemented with Pi is similar to that in TGY control medium, occurring at the end of the exponential and beginning of the stationary phase growth phases. Expression of *tetR* was similarly increased in cultures supplemented with P_i_, but mainly within a shorter time duration between 24 and 32 h of culture (Figure 6E). This suggests that regulation of TeNT expression by inorganic phosphate is independent of *tetR*. 

### 2.7. Carbonate Stimulates TeNT Synthesis

**Figure 7:**
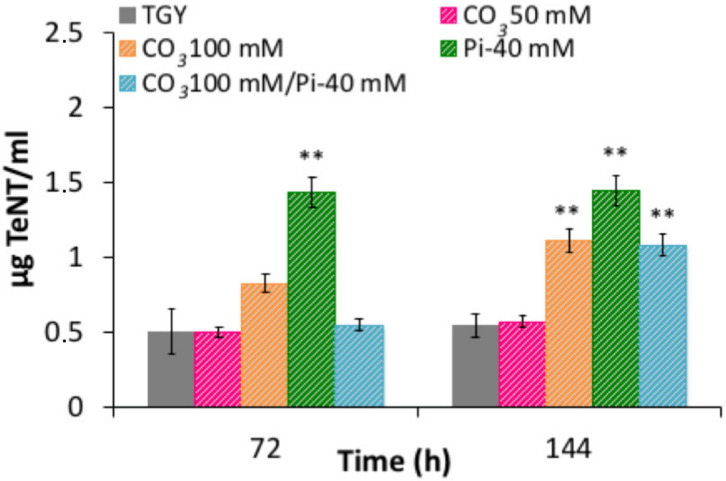
Effects of sodium carbonate and inorganic phosphate on extracellular tetanus toxin (TeNT). *C. tetani* CN655 was grown in TGY supplemented with either 50 or 100 mM Na_2_CO_3_, 40 mM P_i_, or 100 mM Na_2_CO_3_ and 40 mM P_i_. Extracellular toxin levels were increased in TGY medium supplemented with 40 mM P_i_ or 100 mM Na_2_CO_3_. The addition of both P_i_ and Na_2_CO_3_ did not result in a synergistic effect on TeNT production. Data are mean values ± SEM of at least three independent assays. **, *p* < 0.05.

High CO_2_ concentrations (70%) have been found to increase the expression of *bont* and the synthesis of BoNT in *C. botulinum* group II in contrast to group I strains, where CO_2_ has no significant stimulatory effect [27,28]. CO_2_ also increases the toxin production in other bacteria such as *Bacillus anthracis, Staphylococcus aureus* and *Vibrio cholerae* [27]. However, the mechanism of the stimulatory effect of CO_2_ is not well defined. Since *C. tetani* is phylogenetically related to *C. botulinum* [29], we tested the influence of carbonate as a nutritional requirement for TeNT synthesis. For that, the TGY medium was supplemented with various Na_2_CO_3_ concentrations, and addition of 50 or 100 mM Na_2_CO_3_ did not modify the growth of *C. tetani* CN655 (data not shown). We observed a significant stimulatory effect on TeNT synthesis in TGY medium supplemented with 100 mM Na_2_CO_3_ (Figure 7). No synergistic or cumulative effect was detected between the addition of carbonate and P_i_ (Figure 7). The pH at 72 h of culture was slightly higher (pH 7.6) in TGY supplemented with 100 mM Na_2_CO_3_ compared to control TGY (pH 7.0) or TGY with 50 mM Na_2_CO_3_ (pH 7.3). However, the pH in TGY cultures supplemented with 40 mM P_i_ or 100 mM Na_2_CO_3_/40 mM P_i_ was not significantly modified (pH 7.0 and 7.4, respectively), suggesting that the role of P_i_ and Na_2_CO_3_ on TeNT synthesis were not pH-dependent.

### 2.8. The TCS CTC_RS05745/CTC_RS05750 is Involved in Bacterial Cell Wall Organization

**Figure 8:**
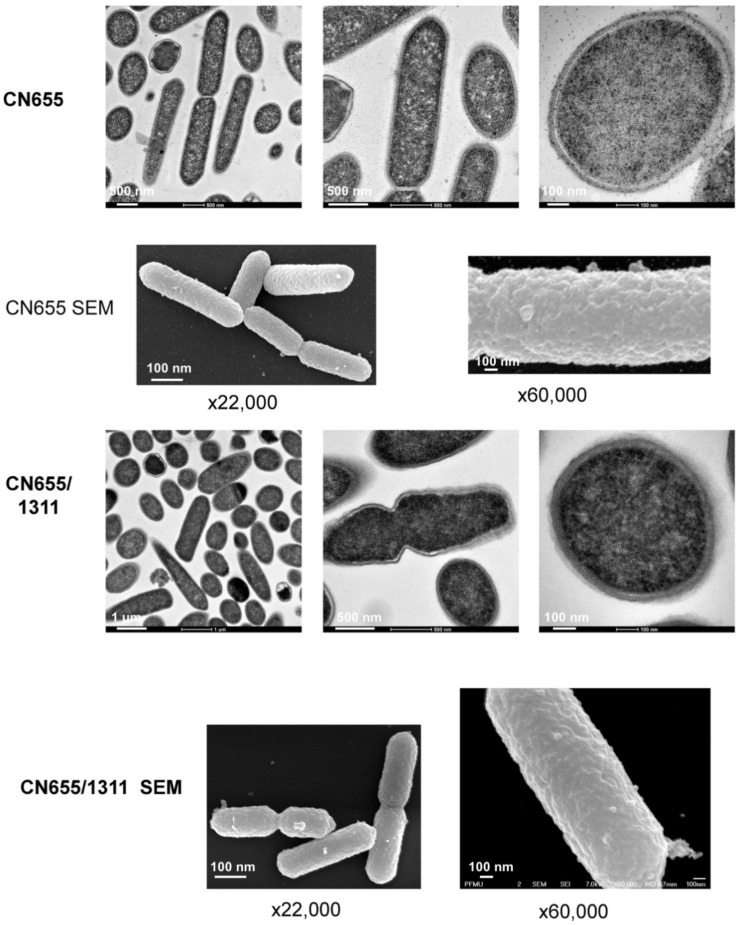
Ultrastructural morphology of CN655 and CN655/p1311. Bacteria from 18 h TGY culture were processed for transmission electron microscopy and scanning electron microscopy (SEM). CN655 showed well-delineated bacterial wall layers, whereas the bacterial wall of CN655/p1311 was disorganized with diffuse and enlarged wall layers. CN655/p1311 showed more abundant blebbings on the bacterial surface. About 100 bacterial cells were observed for each preparation.

Since the TCS CTC_RS05745/CTC_RS05750 seemed to be involved in TeNT secretion without controlling the synthesis level of toxin (see above and Figure 2), we investigated the morphology of the strain CN655/p1311 in comparison to the wt CN655 by electron microscopy (Figure 8). Electron microscopic analysis of wt CN655 and strain CN655/p1311 showed a marked alteration of the bacterial wall of the mutant strain (Figure 8). In the wt CN655 strain, the layers forming the bacterial wall are well organized. In contrast, in strain CN655/p1311, the bacterial wall appeared disorganized and an abundantly detected diffuse material surrounded the bacteria. The altered bacterial wall of CN655/p1311 might account for the increased extracellular TeNT level. 

## 3. Discussion

The mode of TeNT synthesis in *C. tetani* is still poorly understood. TeNT is usually produced by culturing *C. tetani* in complex media, and variability in TeNT production is a major concern in industrial processes. Carbon and nitrogen sources, notably peptides and amino acids, are important parameters for *C. tetani* metabolism and TeNT synthesis [6,30,31,32]. Toxin production in clostridia is typically a complex regulated process [33,34]. The gene *tetR*, which lies directly upstream of *tent*, was the first regulatory element identified in the regulation of TeNT production. The *tetR* gene encodes an alternative sigma factor that positively regulates the transcription of *tent* at the transition phase between the end of the exponential and the beginning of the stationary growth phases [13]. The genome of *C. tetani* strain E88 was found to contain at least 127 genes that encode putative transcriptional regulators, including 30 TCSs and 29 sigma factors [35]. However, their role in the regulation of TeNT synthesis is not yet known. Here, we have investigated 12 putative regulators. These have been selected based on their homology with regulators that have already been identified to be involved in the toxin regulation in other clostridia such as *C. botulinum* and *C. difficile*. 

**Figure 9:**
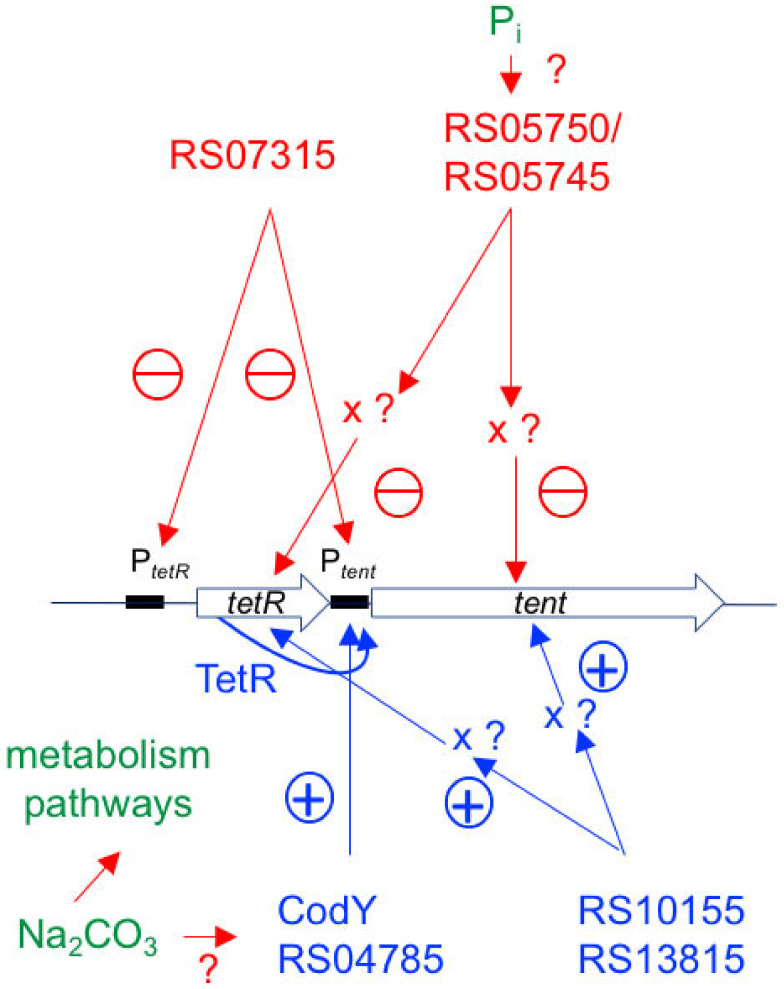
Schematic summary of the regulators of tetanus toxin (TeNT) synthesis of this study. Two two-component systems (TCSs) (RS13815 and RS10155) as well as CodY are positive regulators. They activate the transcription of *tetR* and *tent*, only CodY acts directly by interacting with the promoter of *tent* (P*_tent_*). In addition, the TCS RS04785 increases the extracellular TeNT level without or only weakly affecting the total synthesis by interacting with P*_tent_*. One TCS (RS07315) is a negative regulator that interacts with (P*_tent_*) and (P*_tetR_*). The TCS RS05750 has a moderate negative effect by weakly and indirectly decreasing *tent* and *tetR* transcription. Inorganic phosphate (Pi) and carbonate are environmental factors that influence TeNT synthesis through the TCS pathway and/or through the general metabolism.

Among the nine TCSs which have been investigated by the RNA anti-sense strategy, two positively regulated the production of TeNT (CTC_13810/CTC_13815 and CTC_RS10150/CTC_RS10155) and one was identified as a negative regulator (CTC_RS07310/CTC_RS07315). Moreover, two other TCSs influenced the extracellular toxin level rather than the total production of TeNT, positively (CTC_RS04780/CTC_RS04785) and negatively (CTC_RS05750/CTC_RS05745) (Figure 9). 

The TCSs CTC_13810/CTC_13805 and CTC_RS10150/CTC_RS10155 influenced the *tent-tetR* expression and subsequent TeNT production. However, *tent* and *tetR* transcription were not decreased in the exponential growth phase despite a reduction of TeNT production. This might result from a delayed effect of the RNA anti-sense system and/or of unproductive RNA hybridized with the anti-sense RNA. They seem to indirectly control the expression of *tent* and *tetR*, since the respective RRs bound neither to P*_tent_* nor P*_tetR_*.

The TCS CTC_RS13810/CT_RS13805 is the only TCS located on the large *tetR-tent* operon-containing plasmid, and it is encoded about 25 kb upstream of *tent*. The CTC_RS13805 RR belongs to the OmpR family (Table 1) and shares 91% amino acid identity with a RR of *Clostridium lundense* but has no homolog in other clostridia or bacteria. 

The chromosomally encoded RR CTC_RS10155 is related to the LytR/AlgR family of regulators and is putatively involved in autolysis. This TCS has homologs in *C. botulinum* and other clostridia. However, the TCS homolog in *C. botulinum* has no influence on BoNT production [25].

The *C. botulinum* TCS CBO0786/CBO0787 was previously found to repress BoNT synthesis [26]. This TCS has a homolog in *C. tetani* (CTC_RS07310/CTC_RS07315) with a 58% identity at the amino acid level of the RR, and it is predicted to be involved in cell division. This *C. tetani* RR was also found to repress TeNT synthesis. Indeed, secreted and total TeNT levels were increased in the anti-sense strain (CN655/p1419). Interestingly, the RR CTC_RS07315 bound to P*_tent_* and P*_tetR_*, resulting in a significant decrease in *tent* expression at the stationary growth phase and a moderate impairment of *tetR* transcription at late exponential and early stationary growth phases. This suggests that the negative effect on *tent* transcription results from a direct interaction with P*_tent_*, possibly independent of *tetR* since the effect on *tetR* transcription was weak. The mode of action of CTC_RS07315 RR is likely similar to that of CBO0786, which binds to the *ntnh-bont/A* and *ha* operon promoters in *C. botulinum* and inhibits their transcription, in a *botR*-independent manner [26].

The TCS CTC_RS05750 also exhibits a negative effect on TeNT secretion, since the respective anti-sense strain (CN655/p1311) showed an increased extracellular TeNT level. The CTC_RS05750 RR belongs to the OmpR family and it is supposed to be involved in the phosphate uptake. CTC_RS05750 moderately inhibits *tent* and *tetR* transcription, probably in an indirect manner since in contrast to the RR CTC_RS07315, no binding of CTC_RS05750 RR to P*_tent_* and P*_tetR_* was observed. This TCS probably controls pleiotropic pathways including the assembly of the bacterial wall. The regulatory pathways controlled by CTC_RS05745/CTC_RS05750 are still unknown and this TCS probably has an indirect role in cell wall synthesis. Indeed, a marked alteration of the bacterial wall was observed in CN655/p1311 compared to wt CN655 (Figure 8). The mechanism of TeNT secretion in *C. tetani* is not yet understood. For several decades, it has been known that *C. tetani* autolysis induces TeNT accumulation in the extracellular medium [36]. Cell wall modification in CN655/p1311 was likely responsible for the increased extracellular TeNT without affecting the total level of TeNT synthesis. CTC_RS05745/CTC_RS05750 is homologous to related TCSs in other clostridia, including *C. botulinum*. However, the *C. botulinum* homolog CLC_2386/CLC_2385 (73% amino acid identity between the corresponding RRs) was not found to be involved in the control of BoNT synthesis [25].

The anti-sense strain CN655/p1310, in which CTC_RS04785 RR was targeted, exhibited a slight decrease in the amount of extracellular TeNT and a marked decrease in *tetR* expression. However, only a slight decrease in *tent* expression was observed, albeit CTC_RS04785 RR interacted with P*_tent_* but not with P*_tetR_*, CTC_RS04785 RR has a 34% amino acid identity with the virulence factor VirR of *C. perfringens*. VirS-VirR controls 147 genes in *C. perfringens* including chromosomally-located toxin genes encoding, for example, perfringolysin and alpha-toxin, and plasmid-located toxin genes such as those encoding the Beta*2* toxin and collagen-adhesin [37,38]. The VirS-VirR system of *C. perfringens* can positively or negatively regulate its target genes. Some of the target genes contain a VirR-binding box on their promoter and are directly controlled, whereas a regulatory cascade including a non-coding RNA (VR-RNA) is used in the regulation of other genes [39]. The CTC_RS04780/CTC_RS04785 TCS of *C. tetani* is possibly also involved in a complex network of positive and negative regulatory pathways in *C. tetani*. Our results suggest that this TCS mainly modulates the secretion, and moderately the synthesis, of TeNT, although CTC_RS04785 was found to bind to the *tent* promoter and to stimulate *tetR* transcription.

CodY is a conserved regulator in Gram-positive bacteria, which notably controls metabolism and virulence [17]. In *C. botulinum* A, CodY positively regulates BoNT/A synthesis through binding to the *ntnh-bontA* operon promoter in a GTP-dependent manner [20]. CodY also activates toxin production and virulence in other Gram-positive bacteria such as *Bacillus anthracis, Bacillus cereus, Listeria monocytogenes* and several *Streptococcus* species [40,41,42,43,44]. However, CodY indirectly represses toxin gene expression in *C. difficile* by interacting with the TcdR promoter [45]. Rapidly metabolizable carbohydrates such as glucose repress toxin production in *C. difficile* [46], and CodY is a negative regulator of toxin synthesis and virulence in this pathogen [47]. In contrast to *C. difficile,* TeNT synthesis in *C. tetani* is not inhibited by glucose. TeNT production is higher when cultivation of *C. tetani* is performed in TGY compared to TY media (Supplementary Materials Figure S5). C*. tetani* CodY shares 81% amino acid identity with that of *C. botulinum* type A strain ATCC3502, in which CodY positively controls BoNT/A synthesis [20]. In analogy to its activity in *C. botulinum*, CodY of *C. tetani* bound to P*_tent_* but not to P*_tetR_*, and enhanced *tent* transcription and subsequent TeNT synthesis. This further supports the interaction between metabolism and toxin production in *C. tetani*. Since CodY plays a key role in the adaptation to starvation [19], it could control TeNT synthesis via sensing the availability of specific nutrients.

Spo0A is also a master regulator that has been initially identified to control the first steps of sporulation, it also regulates numerous other genes, notably those involved in adaptation to environmental conditions [48]. For example, Spo0A is involved in the switch between the acidogenesis and solventogenesis/sporulation pathways in environmental bacteria such as *Clostridium acetobutylicum* [49]. Here, Spo0A was not found to control TeNT synthesis, in contrast to its positive impact on BoNT synthesis in *C. botulinum* E through binding to the *bontE* promoter [50]. It is noteworthy that *C. botulinum* E gene cluster does not contain any *botR* or *tetR* homologs in contrast to *C. tetani* and *C. botulinum* group I neurotoxin gene clusters. It is questionable whether there is an interplay between Spo0A, BotR/TetR and toxin synthesis. Spo0A controls the sporulation in *C. botulinum* [23], but we observed no correlation between sporulation and toxin production in *C. botulinum* A [51]. *C. tetani* CN655 formed no spore in TGY, albeit this strain synthesized TeNT. CN655 sporulates only poorly (10–100 spores/ml) in the biphasic medium of Anellis et al. [52], and spores were not detected in CN655/*spo0A* cultures in the biphasic medium, indicating that the anti-sense *spo0A* was functional in CN655. In *C. difficile*, Spo0A differently modulates the toxin synthesis, which depends on the genetic background. In certain *C. difficile* strains such as ribotype 027 strains, Spo0A negatively regulates the production of Toxin A and Toxin B, whereas in other strains, Spo0A has no effect on toxin production [53].

In *C. difficile*, the protein Mfd is involved in nucleotide excision repair and transcription elongation. This protein has been identified as a positive regulator of Toxin A and Toxin B synthesis. Mfd possibly prevents the inhibitory effect of CodY and CcpA (carbon catabolite protein A) by inhibiting their binding to the toxin gene promoters [24]. Mfd is conserved in *C. tetani*. However, it had no impact on TeNT synthesis under the conditions tested. In contrast to *C. difficile*, TeNT synthesis is not repressed by rapidly fermentable carbohydrates [54].

Albeit numerous regulatory genes are conserved in clostridia, they have distinct pleiotropic effects in each species. Our data indicate that *C. tetani* retains unique regulatory pathways to control TeNT synthesis, possibly as an adaptation to specific nutrients and/or environmental conditions.

The complex network of toxin regulation in *C. tetani* raises the question of the environmental factors that trigger the different regulatory components of the network. Since some TCSs are putatively involved in P_i_ uptake and/or metabolism, we tested the influence of P_i_. We observed that an optimum P_i_ concentration around 40 mM significantly increased the transcription of *tent* and subsequent production of TeNT. In *C. perfringens*, 20 to 50 mM P_i_ concentrations enhance the sporulation and production of *C. perfringens* enterotoxin, the gene expression of which is dependent on sporulation. P_i_ induces *spo0A* expression and subsequent sporulation [55,56]. However, we observed no impact of Pi supplementation on sporulation of CN655 in TGY medium. P_i_ was also found to increase the toxin production in *Bacillus thuringiensis* [57]. P_i_ is a key element in bacterial metabolism; notably, it is incorporated into transcriptional regulatory proteins, and P_i_ homeostasis has a critical adaptive role in bacterial virulence [58]. For example, P_i_ availability controls motility, biofilm formation, colonization, induction of virulence factors such as C phospholipases in *Pseudomonas aeruginosa*, colonization factor and toxin synthesis in *Vibrio cholerae*, enteropathogenic and enterohemorrhagic *Escherichia* coli (review in Reference [58]). In *Bacillus anthracis,* P_i_ starvation enhances spore germination, invasiveness in macrophages and toxin secretion [59]. It can be speculated that P_i_ availability might also facilitate wound colonization by *C. tetani* and in situ TeNT production. The mechanism of P_i_ in the control of *tent* transcription is not yet known. It is possibly mediated by TCS such as CTC_RS05745/CTC_RS05750 and CTC_RS10030/CTC_RS10035, which are putatively involved in phosphate uptake. Indeed, the corresponding anti-sense strains (CN655/p1311 and CN655/p1313) grown in TGY supplemented with P_i_ (40 mM) showed slightly increased extracellular TeNT compared to culture in TGY (Supplementary Materials Figure S6). However, these two TCSs might have pleiotropic effects and indirectly control TeNT synthesis, since they are homologs of the PhoP/PhoR TCS which has been found to control diverse metabolism pathways such as redox homeostasis and adaptation to acidic pH in *Mycobacterium*, as well as primary and secondary metabolism pathways under phosphate limitation [60,61]. 

CO_2_ at high concentration (70%) in the gas phase stimulates *bont* gene expression and BoNT production, despite a reduced growth rate of *C. botulinum* group II strains, in which the regulatory *botR* gene is missing. The mode of action of CO_2_ is still speculative: it is supposed to dissolve and increase the bicarbonate concentration in the TGY culture medium and subsequently to enhance carboxylation reactions [62]. Increased bicarbonate concentrations were also found to enhance toxin production in *C. difficile,* possibly through biotin-dependent carboxylation [63]. In our conditions, the extracellular TeNT levels were increased in TGY media containing 100 mM Na_2_CO_3_ in a pH-independent manner, whereas no significant difference was observed with 50 mM Na_2_CO_3_ supplementation. The involvement of carbonate in carboxylation reactions is more likely responsible for the regulatory effects on TeNT synthesis than the buffer effect of carbonate.

In conclusion, TeNT synthesis in *C. tetani* is under the control of a complex network of regulatory elements, including TCSs and regulators of metabolism, as tested by the anti-sense RNA system. However, a more direct approach by deletion of the putative target regulatory genes is required to confirm the regulatory function on TeNT synthesis of these genes. *C. tetani* and *C. botulinum* are related clostridial species which synthesize potent neurotoxins in a regulated manner, and their genomes contain numerous TCSs genes (30 in *C. tetani* and 39 in *C. botulinum* A [25,35]). Interestingly, albeit most of TCSs genes are homologous in both species, *C. tetani* and *C. botulinum* mainly use distinct sets of TCSs in the regulation of toxin synthesis. Among the nine RRs investigated in *C. tetani*, only one system (CTC_RS07315 in *C. tetani* and the homolog CBO0786 in *C. botulinum*) has a similar function in both species, i.e., serves as a negative regulator of toxin synthesis [26]. Four other RRs modulate TeNT synthesis (one acting as negative and three as positive regulators), whereas their homologous counterparts in *C. botulinum* A have no effect on toxin production [25]. In addition, two TCSs, which positively control BoNT production in *C. botulinum*, have homologs in *C. tetani* that do not impact on TeNT synthesis. We hypothesize that *C. tetani* has adapted TeNT synthesis to specific nutritional and environmental requirements. Indeed, *C. tetani* preferentially uses specific metabolic pathways [35]. Notably, peptides and amino acids are critical substrates for *C. tetani* and specific short peptides from casein digestion are essential for TeNT synthesis [32,35]. Here, we also found that P_i_ and carbonate are additional nutritional requirements for TeNT production. Thereby, TeNT synthesis is dependent of a complex network of regulation linked to *C. tetani* metabolism, in which carbohydrates and P_i_ are important elements. A better understanding of the regulation of TeNT synthesis and the underlying environmental factors is required to optimize the toxin production by *C. tetani* for vaccine production. Albeit tetanus is rare in the developed world, it is still an important cause of deaths in many underdeveloped countries, with 34,000 estimated neonatal tetanus deaths in 2015 [64]. Immunization against TeNT is an efficient method of tetanus prevention, and thus, a 96% reduction of this disease was achieved since 1988 [64]. Availability of a cheap and efficient tetanus vaccine is an important factor for the eradication of this disease. Unraveling the regulation of TeNT synthesis constitutes the basis for constructing hyperproductive strains.

## 4. Materials and Methods 

### 4.1. Bacterial Strains and Culture Conditions 

The recombinant strains used in this study are presented in Table 2. *Escherichia coli* strains were grown in Luria-Bertani (LB) broth and *C. tetani* CN655 in TGY broth (pH 7.5) containing trypticase Peptone (BD Biosciences; 30 g/L), yeast extract (Bacto Yeast Extract, BD Biosciences; 20 g/L), glucose (5 g/L) and cysteine, HCl (0.5g/L) under anaerobic conditions (N_2_/CO_2_/H_2_; 90: 5:5, vol/vol.) at 37 °C. When necessary, erythromycin was added to culture media at 5 or 50 µg/ml for *C. tetani* and 300 µg/mL for *E. coli*. *C. tetani* was grown in TGY with different inorganic phosphate (P_i_) concentrations by the addition of Na_2_HPO_4_, and/or with sodium carbonate Na_2_CO_3_ (Merck, Guyancourt, France). 

Growth kinetics of *C. tetani* CN655 strains in TGY broth culture medium supplemented with erythromycin (5 μg/mL) were monitored by spectrometry at 600 nm over a 144 h period.

### 4.2. Construction of Vectors Encoding Anti-Sense mRNA for the Different Two-Component Systems (TCS) and Others Regulators 

A DNA fragment of each TCS and regulatory genes studied containing the ribosome binding site (RBS) region was amplified by PCR and inserted in reverse orientation into pAT18, as already described in Reference [14]. DNA segments for anti-sense mRNA production were designed in the RR of three TCS, in the SHK gene of six TCS, as well as in three regulatory genes (*codY*, *spo0A* and *mfd*) (Table 2). The PCR products from CN655 genome DNA contain a 3’*Nco*I site and a 5’ *Pst*I site and were cloned into pMRP306, a derivative of pAT19 containing the promoter of the iota toxin gene, the cloning sites *Nco*I-*Pst*I and the 3’ part of the iota toxin gene [14]. The resultant anti-sense RNA plasmids were transformed into CN655 by electroporation.

The recombinant plasmids were prepared in *Escherichia coli* Top10 strain (Dam+, Dcm+) and electroporated into *C. tetani* CN655, with an efficiency of 25–50 transformants per 1 μg DNA. Interestingly, no transformant was obtained from plasmids prepared in *E. coli* BL21 (Dam+, Dcm−) or C2925 (Dam−, Dcm−), indicating that *C. tetani* CN655 contains a functional Dcm methylase that impairs the transformation of non-DCM-methylated DNA.

### 4.3. Tetanus Toxin Assay

At 8, 24, 32, 48, 56, 72 and 144 h of culture growth, 4 mL of the culture were removed. The cells were harvested at 10000 rpm for 10 min at 4 °C, and the supernatants corresponding to the extracellular toxin were filtered (0.22 μm) and stored at −20 °C. For intracellular toxin assay, the pellets were washed twice with distilled water and two osmotic lysis were with TGY containing 20 mg/mL NaCl and 13.3 mg/mL sodium citrate (C_6_H_5_Na_3_O_7_, 2H_2_O) during 24 h, at 4 °C. After centrifugation, the supernatants containing intracellular toxin were filtered and stored at −20°C.

Extracellular and intracellular toxin were monitored by an enzyme-linked immunosorbent assay (ELISA). Wells of microtiter plates (Nunc Maxisorp; Nunc, Roskilde, Denmark) were coated with 100 µL of equine anti-tetanus serum (Sanofi Pasteur, FA193727) in 0.05 M carbonate buffer pH 9.5 and incubated at 4 °C overnight. Plates were washed 3 times with phosphate buffered saline (PBS)-Tween20 0.1% with an automatic plate washer (BioTek, Washer 120, BioTek France, Colmar, France). After blocking with 20 mg/ml BSA in carbonate buffer during 30 minutes under agitation, three washes were performed, and 100 µl/well of serial two-fold dilutions in PBS-Tween20 0.1%-BSA 1% (PBS-T-BSA) of samples were added. The plates were incubated for 1 h at room temperature with shaking. Tetanus toxin (Sanofi Pasteur, Marcy l’Etoile, France) was used as standard. After three washes, the plates were incubated with 100 μL/well of rabbit anti-tetanus serum (1:6400; Sanofi Pasteur n° 7078) for 1 h at room temperature, then with 100 µl of goat anti-rabbit Ig peroxidase-linked (1:4000, 111-035-006, Jackson Immunoresearch) for 1 h at room temperature in PBS-T-BSA. For detection, 100 µL of 1 mg/mL ortho-phenylene-diamine (OPD, Sigma) in citrate buffer (0.05 M, pH 4.5 containing 0.06% H_2_O_2_) was used. The color development was stopped after 8 min by adding 50 µL 3 M HCl. The absorbance was read on a microplate reader (Biorad, model 680) at 490 and 655 nm. 

### 4.4. Total RNA Extraction, Reverse Transcription and Quantitative Real-Time PCR Assay

Total RNA from *C. tetani* strains were extracted at 8, 24, 32 and 48 h of growth. After centrifugation at 4000 rpm for 15 min at 4 °C, the culture pellet was mechanically disrupted in the presence of silica beads (Lysing Matrix B, MP Biomedicals, Illkirch, France) and buffer RLT (MP Biomedicals) by shaking with a FastPrep apparatus (MP Biomedicals), and RNA total extracted using RNeasy mini kit (Qiagen, Courtaboeuf, France), according to the manufacturer’s instructions. The RNA preparations were stored at −80 °C. 

A DNAse treatment with TURBO DNase (Ambion, Thermoscietific, Les Ulis, France was performed following the manufacturer’s instructions. The absence of DNA contamination on RNA extracts was checked by real-time PCR (RT-PCR) targeting *tent*. Total RNA amount was monitored with NanoDrop ND-100 Spectrophotometer. cDNAs were then synthesized from 1 µg of total RNA with random primers (pDN6 5 µg/µL, Roche, Meylan France) and RNase OUT™ Recombinant Ribonuclease Inhibitor (Invitrogen, ThermoFischer, Les Ulis, France) and with M-MLV Reverse Tanscriptase kit (Invitrogen), according to the manufacturer’s instructions. 

RT-PCR was performed in 25 µl reaction volume containing 30 ng of cDNAs, 12.5 µL of SYBR Green Supermix (Bio-Rad, 2×; 1.25 U iTaq DNA polymerase, 0.4 mM each dNTP, 6 mM MgCl2, 20 nM fluorescein, SYBR Green I) and 500 nM gene-specific primers (Table 3) in an iQ iCycler apparatus (Bio-Rad). The reaction was subjected to denaturation at 95 °C for 3 min followed by 40 cycles of denaturation at 95 °C for 10 s, annealing/elongation at 61.7 °C for 30 s for *rpoB* and *tent* genes and 65.1 °C for *gyrA* and *tetR* genes. Then, a dissociation stage of 65 to 95 °C with a heating rate of 0.5 °C per 10 s was performed to establish a melting curve to confirm the specificity of the RT-PCR reaction for each primer pair.

The relative cDNA quantity of each sample was determined with the threshold cycle [ΔΔCT] method (Analysis of Relative Gene Expression Data Using Real-Time Quantitative PCR and the 2ΔΔCT method). cDNA quantity of the *tent* and *tetR* gene was normalized to the quantity of cDNA of the *rpoB* and gyrA gene.

### 4.5. Expression and Purification of Recombinant Proteins 

The genes *ctc_p21, ctc_01979, ctc_1421, ctc_00935*, *codY* and *tetR* were PCR-amplified from the genome of *C. tetani* CN655, with primers (Table 3), adding a *Bam*HI site at the 5’end and an *Eco*RI at the 3’ end. PCR products were digested with appropriate restriction enzymes and the resulting products were cloned into a pCR2.1 vector and subcloned into pET28a (Novagen, Merck, Guyancourt, France) for the expression of N-terminal 6-histidine proteins. The resulting constructions were transformed in *E. coli* BL21DE3, according to the manufacturer’s instructions. 

Recombinant proteins were purified by affinity chromatography employing the TALON cobalt affinity resin (Clontech Laboratoires, Saint Germaine n Laye, France) in accordance with the standard protocol provided by the manufacturer. Briefly, to induce expression of recombinant proteins, clones were grown in 1 litter of LB supplemented with 50 µg/mL kanamycin at 37 °C to an optical density at 600 nm of 0.6–0.8. Protein expression was then induced by the addition of isopropyl-β-D-thiogalactopyranoside (IPTG) at a final concentration of 0.5 mM and the growth was continued overnight at 18 °C. The bacteria were collected by centrifugation, suspended in PBS pH 8.0 containing 10 mM imidazole and protease inhibitors (EDTA free protease inhibitor cocktail, Roche), and lysed by sonication. The recombinant proteins were eluted with 100 mM imidazole and dialyzed overnight against PBS and then frozen at −80 °C for storage. Quantification of the protein was done using the Bradford reagent (BioRad, Marnes La Coquette, France), following the manufacturer’s instructions. Aliquots of fractions were analyzed on 12% sodium dodecyl sulfate-polyacrylamide gel electrophoresis (SDS-PAGE).

### 4.6. Electrophoretic Mobility Shift Assay (EMSA)

A 359 bp fragment covering *tent* promoter (P*_tent_* probe) and a 316 bp fragment covering *tetR* promoter (P*_tetR_*) were amplified by PCR using 5’-end biotin-labeled primers (Table 3). Binding reactions were carried out for 1 h at room temperature using 5 μM of recombinant proteins, 5’-end biotin-labeled probe at 0.2 nM for P*_ten_t* and 0.1 nM for P*_tetR_*, 50 ng/μL of poly (dI-dC), 2.5% glycerol and 5 mM MgCl_2_ in binding buffer. Competition assays for binding specificity were performed with a 300-fold excess of unlabeled specific probe. 

Reactions were separated on a 5% native polyacrylamide gel, run in 0.5× Tris-borate buffer-EDTA (TBE) at 4 °C for 1 h at 110 V and electrotransferred to a positively charged nylon membrane (Amersham) at 380 mA for 30 min. Transferred DNAs were cross-linked to membranes with UV light. The biotin-labeled DNAs were detected with the LightShift Chemiluminescent EMSA kit (Pierce, ThermoFischer, Les Ulis, France) according the manufacturer’s specifications. 

### 4.7. Electron Microscopy

Bacteria from 18 h culture were mixed V/V with a fixative solution containing 5% glutaraldehyde (GA) in 0.2 M PHEM buffer pH 7.2 (60 mM Pipes, 25 mM Hepes, 10 mM EGTA, 2 mM MgCl_2_) and incubated for 1 h. Samples were washed twice in PBS prior to performing a high-pressure (>2000 bar) freezing in 1-hexadecane using a BAL-TEC HPM 010 (LEICA). Freeze substitution was done with 2% OsO_4_ and 0.5% Uranyl Acetate in acetone followed by several steps: −90 °C for 42 h, warmed up to −30 °C (5 °C/h), incubation for 12 h, warmed up to 0 °C (10 °C/h) and incubation for 1 h. Then, samples were washed with acetone on ice and incubated with increasing low-viscosity embedding media SPURR’s kit (EMS ref. 14300)/acetone mixture (1:4) for 3 h, 1:1 over-night, 3:1 during the day, 9:1 over-night, SPURR resin for the day and over-night. The samples were then placed into flat tubes infiltrated with pure SPURR resin prior to polymerization at 60 °C for 48 h. Sections (60–70 nM) were obtained on a FC6/UC6 ultramicrotome (Leica, Wetzlar, Germany), transferred on 200 Mesh Square Copper grids coated with formvar and carbon (CF-200-Cu50, Delta Microscopy). Samples were stained with 4% uranyl acetate and counterstained with lead citrate. Images were recorded with TECNAI SPIRIT 120 kv (with a bottom-mounted EAGLE 4K x 4K Camera, ThermoFisher Scientific, Waltham, MA, USA).

For scanning electron microscopy (SEM), the samples dehydrated with ethanol were dried with CO_2_, and then sputtered with 20 nm gold palladium with a GATAN Ion Beam Coater and were examined with a JEOL JSM 6700F field emission scanning electron microscope operating at 7 Kv. Images were acquired with the upper SE detector (SEI).

### 4.8. Statistical Analysis

Values throughout are expressed as means ± standard error of the mean. Differences in the different anti-sense strains were assessed using unpaired Student’s t-test, where statistical significance is assumed for * *p* < 0.05, ** *p* < 0.01 and *** *p* < 0,001.

## Figures and Tables

**Table 1:** Two component system (TCS) genes encoded in the genome of *C. tetani* E88 and their homology with regulatory genes in other clostridia. The recombinant RNA antisense plasmids targeting TCS genes are indicated in column 1. Genetic environment indicates functional genes in close proximity of TCS in *C. tetani* E88.

Recombinant Antisense mRNA Plasmid	Gene Bank Accession Number		Other Gene Name	Role	Familly (RR)	Genetic Environment	Homology	Homologs (RR) in Other Clostridia (Protein Identity > 60%)	Homolog TCS in *C. botulinum strain* Hall			Homolog TCS in *C. botulinum* Strain ATCC 3502	
	Old Locus Tag	Locus Tag							Homolog System (RR/SHK)	Protein Identity (RR)	Regulation of Botulism Neurotoxin	Homolog System (RR/SHK)	Protein Identity/Positive
	**chromosomal localization**												
	CTC_00189	CTC_RS00820		RR	OmpR	Periplasmic endopeptidase	ResD, respiration control	*C. botulinum, C. cochlearium, C. tetanomorphum, C. scatologenes, C. drakei, C. magnum, C. sporogenes, C. tyrobutyricum, C. acetireducens, C. ljungdahlii, C. novyi, C. pasteurianum,*	CLC_3521/CLC_3520	81%	None	CBO3543	81/87
	CTC_00191	CTC_RS00825		SHK								CBO3542	67/73
	CTC_00392	CTC_RS01985		RR	OmpR	ABC Transporter	BacS/BacR regulation of resistance to bacitracin	*C. botulinum, C. tetanomorphum, C. sporogenes, C. lundense, C. cavendishii, C. acetobutylicum, C. pasteurianum, C. ljungdahlii*	CLC_0331/CLC_0332	90%	None	CBO0272	90/92
	CTC_00393	CTC_RS01990		SHK								CBO0273	86/89
	CTC_00411	CTC_RS02080	phoP	RR	OmpR	hydroxylamne reductase	VirI/VirJ regulation of toxin synthesis	*C. botulinum, C. perfringens, C. carboxidivorans, C. pasteurianum, C. lundense, C. novyi, C. Septicum*	CLC_0661/CLC_0663	65%	Positive	CBO0607	64/72
p1308	CTC_00412	CTC_RS02085	phoR	SHK								CBO0608	46/55
	CTC_00455	CTC_RS02330		RR		phosphomannomutase/ phosphoglucomutase	putative rRNA methylase	*C. tetanomorphum, C. ljungdahlii, C. homopropionicum*	CLC_0413	55%	unknown	CBO0355	54/59
	CTC_00456	CTC_RS14405		SHK								no	
	CTC_00597	CTC_RS02990		RR	OmpR	Glycerine-deshydrogenase	BacR regulation of resistance to bacitracin	*C. lundense, C. tetanomorphum, C. ljungdahlii, C. argetinense, C. drakei*	CLC_2212/CLC_2211	45%	None	several	45/66
	CTC_00598	CTC_RS02995		SHK								no	
	CTC_00628	CTC_RS03125	spaR	RR	OmpR	ABC Transporter SpaEFG: export subtiline	SpaR/SpaK regulation of resistance to subtilin	*C. botulinum, C. novyi, C. beijerinckii, C. saccharobutylicum, C. butyricum, C. sporogenes*	CLC_1615/CLC_1616	65%	unknown	CBO1585	65/69
	CTC_00629	CTC_RS03130	spaK	SHK								CBO1586	37/43
	CTC_00805	CTC_RS04010		RR	OmpR	Protein transport	VanR/VanS regulation of resistance to vancomycin	*C. botulinum, C. cochlearium, C. tetanomorphum, C. lundens, C. carboxidivorans, C. sporogenes, C. pasteurianum*	CLC_0423/CLC_0424	76%	None	CBO0365	78/82
	CTC_00806	CTC_RS04015		SHK								CBO0366	44/50
	CTC_00848	CTC_RS04235		RR	OmpR	Heat shock protein	GtcS/GtcR regulation of antibiotic synthesis (gramicidin)	*C. lundense, C. tetanomorphum, C. amylolyticum, C. perfringens*	CLC_1640/CLC_1639	48%	None	several	47/55
	CTC_00849	CTC_RS04240		SHK								no	
	CTC_00872	CTC_RS04365		RR	OmpR	Efflux-ATPase Copper	Arc, regulation of aerobic/anaerobic respiration	*C. botulinum, C. tetanomorphum, C. lundense, C. sporogenes, C. pasteurianum, C. beijerinckii,, C. ljungdahlii*	CLC_1088/CLC_1089	68%	None	CBO1035	66/71
	CTC_00873	CTC_RS04370		SHK								CBO1036	55/63
	CTC_00924	CTC_RS04645		SHK	PucR	Zn-dependent protease	regulation of purine catabolism, methyl-accepting chemotaxis protein		No			CBO1773	43/50
	CTC_00925	CTC_RS04650		RR								no	
p1309	CTC_00934	CTC_RS04705		SHK	AraC	amihydrolase; Pyruvate formate lyase	LytS, autolysis regulation	*C. botulinum B str. Eklund 17B, C. novyi*	CLC_1627	41%	unknown	no	
	CTC_00935	CTC_RS04710		RR								no	
	CTC_00949	CTC_RS04780	virS	SHK	LytR/AlgR	transcriptional regulator, merR family; putative methyl-accepting chemotaxis protein	virulence regulation	*C. butyricum, C. septicum*	CLC_1105/CLC_1104	35%	None	no	
p1310	CTC_00950	CTC_RS04785	virR	RR								CBO1053	35/54
	CTC_01130	CTC_RS05745		RR	OmpR	ABC Transporteur: phosphates	PhoP/PhoR, regulation of phosphate uptake	*C. lundense, C. carboxidivorans, C. ljungdahlii, C. botulinum, C. sporogenes, C. butyricum, C. acetobutylicum, C. butyricum, C. pasteurianum, C. neonatale, C. baratii*	CLC_2386/CLC_2385	73%	None	CBO2527	73/87
p1311	CTC_01131	CTC_RS05750	phoR	SHK								CBO2526	52/75
p1312	CTC_01211	CTC_RS06180	tlpA	SHK	LysR	anaerobic sulfite reductase	LytR, autolysis regulation, methyl-accepting chemotaxis protein tlpA	*C. cochlearium*	CLC_3570	35%	unknown	CBO2828	36/61
	CTC_01212	CTC_RS06185		RR								several	35/57
	CTC_01420	CTC_RS07310	resE	SHK	OmpR	ABC Transporter	YycG/YycF, regulation of cell division	*C. lundense, C. cellulovorans, C. amylolyticum, C. botulinum,*	CLC_0842/CLC_0843 CB00786/CB00787 *	58%	None Negative	CBO0787	47/65
p1419	CTC_01421	CTC_RS07315		RR								CBO0786	58/76
	CTC_01481	CTC_RS07700		SHK	OmpR	Conserved proteins with transmembrane helices and 4Fe4S motif	FeuQ/FeuP, regulation of iron acquisition	*C. novyi, C. yurii*	CLC_3521/CLC_3520	43%	None	no	
	CT_01482	CTC_RS07705		RR								several	43/60
	CTC_01490	CTC_RS07755		RR	OmpR	Heat shock protein HtpG (chaperonne); Membrane protein	unknown	*C. lundense, C. tetanomorphum, C. kluyveri, C. pasteurianum, C. carboxidivorans, C. ljungdahlii, C. tyrobutyricum, C. acetobutylicum*	CLC_0423/CLC_0424	44%	None	several	44/64
	CTC_01491	CTC_RS07760		SHK								no	
	CTC_01523	CTC_RS07895		RR	NarL	Fumarate-reductase soluble flavoprotein	DcuR, regulation of fumarate anaerobic respiration through C4-dicarboxylates	*C. cochlearium*	CLC_0307/CLC_0306	48%	None	CBO0249	48/69
	CTC_01524	CTC_RS07900	dpiB	SHK								CBO0248	41/60
	CTC_01804	CTC_RS09305		SHK	OmpR	ABC Transporter	BacS/BacR, AB-Bacitracine synthesis and regulation	*C. tetanomorphum, C. lundense, C. novyi*	CLC_2212/CLC_2211	56%	None	CBO2284	47/72
	CTC_01805	CTC_RS09310		RR								CBO2285	56/79
	CTC_01818	CTC_RS09380	resE	SHK	OmpR	ABC Transporter; RNA polymerase sigma factor	unknown	*C. tetanomorphum, C. lundense, C. ljungdahlii, C. cavendishii*	CLC_0842/CLC_0843 CB00786/CB00787*	51%	None Negative	CBO0787	38/63
	CTC_01819	CTC_RS09385		RR								CBO0786	51/71
	CTC_01848	CTC_RS14320	yesM	SHK	NarL/FixJ	Fumarate-reductase	unknown	*C. cochlearium*	CLC_2236/CLC_2235	26%	None	no	
	CTC_01849	CTC_RS09510		RR								no	
	CTC_01857	CTC_RS09550		SHK	XRE	Helicase, oleate hydratase	SinR regulation of entry in stationary phase, to nutrient depletion; Spo0A repressor	*C. botulinum CDC_69094, C. magnum, C. beijerinckii*	No			CBO0693	48/70
	CTC_01858	CTC_RS09555	sinR	RR								no	
	CTC_01905	CTC_RS09790		SHK	OmpR	ABC Transporter	BacR; VanR (synthèse et régulation AB)	*C. indolis, C. methoxybenzovorans*	CLC_0423/CLC_0424	45%	None	no	
	CTC_01906	CTC_RS09795		RR								CBO0365	45/66
	CTC_01918	CTC_RS09860	resE	SHK	OmpR	ABC Transporter	BacR; VanR (synthèse et régulation AB)	*C. kluyveri, C. uliginosum, C. puniceum, C. scatologenes, C. saccharobutylicum, C. oryzae, C. ljungdahlii, C. lundense, C. drakey, C. botulinum B2 331, C. sporogenes, C. acetobutylicum, C. butyricum*	CLC_0842/CLC_0843 CB00786/CB00787*	42%	None Negative	CBO0787	32/53
	CTC_01919	CTC_RS09865		RR								CBO0786	42/60
p1313	CTC_01951	CTC_RS10030	phoR	SHK	OmpR	Heavy metal translocating P-type aTPase	PhoP/PhoR regulation of phosphate uptake	*C. cochlearium, C. lundense, C. tetanomorphum, C. intestinale, C. amylolyticum, C. baratii, C. botulinum, C. chauvoei, C. sporogenes, C. perfringens, C. ljungdahlii*	CLC_0410/CLC_0411	68%	Positive	CBO0353	57/78
	CTC_01953	CTC_RS10035	phoP	RR								CBO0352	68/86
	CTC_01978	CTC_RS10150		SHK	LytR/AlgR	carbon starvation protein A CstA	LytS/LytR autolysis regulation	*C. cochlearium, C. tetanomorphum, C. lundense*	CLC_3250/CLC_3251	55%	None	CBO3309	51/71
p1314	CTC_01979	CTC_RS10155		RR								CBO3308	55/78
	CTC_02155	CTC_RS11115		SHK	OmpR	DNA mismatch repair protein hexA	VanS/VanR regulation of resistance to glycopeptides	*C. carboxidivorans, C. lundense, C. amylolyticum, C. botulinum, C. oryzae, C. pasteurianum, C. sporogenes, C. novyi, C. neonatale*	CLC_1640/CLC_1639	77%	None	CBO1612	48/68
	CTC_02156	CTC_RS11120		RR								CBO1613	77/86
	CTC_02178	CTC_RS11240		SHK	Fis	ethanolamine utilization protein EutA, EutP	EutS/EutR regulation of éthanolamine utilization	*C. argentinense, C. drakei, C. lundense, C. tetanomorphum*	No			no	
	CTC_02179	CTC_RS11245		RR								no	
	CTC_02322	CTC_RS11915		RR	Fis	sodium/glutamate symport carrier protein; V-Typ-ATPase-protein	AtoS/AtoC regulation of acetoacetate metabolism	*C. cochlearium, C. lundense, C. tetanomorphum*	CLC_1882	40%	unknown	several	46/63
	CTC_02323	CTC_RS11920		SHK								no	
	**plasmid localization**												
p1307	CTC_p22	CTC_RS13810		SHK	OmpR	ATP-binding protein	unknown	*C. lundense*	CLC_1431/CLC_1432	56%	None	CBO1395	39/60
	CTC_p21	CTC_RS13805		RR								CBO1394	56/74

RR, Response Regulator; SHK, Sensor Histidine Kinase, * 100% homology with TCSs of *C. botulinum* ATCC3502.

**Table 2:** CN655 anti-sense strains, and primers used for the construction of recombinant plasmids generating antisense mRNA. The PCR products from CN655 genome DNA contain a 3’*Nco*I site and a 5’*Pst*I site and were cloned into pMRP306, a derivative of pAT19 containing the promoter of the iota toxin gene, the cloning sites *Nco*I-*Pst*I and the 3’part of the iota toxin gene [14]. The resultant antisense RNA plasmids were transformed into CN655 by electroporation. SHK: Sensor Histidine Kinase, RR: Response regulator.

Isogenic Antisense Strains	Target Gene	S/R	Primer	Nucleotide Sequence (5′--> 3)	Product Length (bp)
CN655/1307	CTC_p22	S	P2020-F	CCGCTGCAGGATAATTTGGGAATGATTATTTTA	228
			P2021-R	GGCCATGGTTAACATATCGTCCATACTC	
CN655/1308	CTC_00412	S	P2022-F	CCGCTGCAGGAGGTGATTGAAAAATAG	208
			P2023-R	GGCCATGGTAAATCTAACATAGTAAATTTATAC	
CN655/1310	CTC_00950	R	P2024-F	CCGCTGCAGGGAGGGTTAAATTATGTATAATG	236
			P2025-R	GGCCATGGGCTACTTCTATACCATTTATTTC	
CN655/1309	CTC_00934	S	P2026-F	CCGCTGCAGCAGGGGGTATTTTTGTGTTAAATAATAGG	236
			P2027-R	GGCCATGGCATTGGCATCGCAACATATGCG	
CN655/1312	CTC_01211	S	P2028-F	CCGCTGCAGGGGGAGACAGTGGTGAAGTTGCG	223
			P2029-R	GGCCATGGGGTTAAAAAATTTTCTTTTATATTTC	
CN655/1314	CTC_01979	R	P2030-F	CCGCTGCAGGAGATGAATTTATGAACAAAAT	231
			P2031-R	GGCCATGGGCTAATTCCATGCCATTTTTAG	
CN655/1311	CTC_01131	S	P2032-F	CCGCTGCAGGGTGGTAAAATGAAAAAAAG	245
			P2033-R	GGCCATGGCCTTATATCACTATCATTA	
CN655/1313	CTC_01951	S	P2034-F	CCGCTGCAGGGAAGGTAGAAAATGAAAAGTATAAAG	243
			P2035-R	GGCCATGGCCACATTATCCATTATATTTTCTTC	
CN655/1419	CTC_01421	R	P2291-F	CCGCTGCAGGGGAGATTTTGTGAACAACATATT	242
			P2292-R	GGCCATGGTCTGATGCCTTTCTTATTTCTTTAC	
CN655/1418	CTC_01260	CodY	P2289-F	CCGCTGCAGGAGGAGTTACAAATGTCATCATTATTA	232
			P2290-R	GGCCATGGACTACCTTGTCTCTTACTGTCTG	
CN655/1472	CTC_00222	Spo0	P2361-F	CCGCTGCAGGGAGGTATAAAATATATGATA	225
			P2362-R	GGCCATGGTTATTACACTCTTTAAAGGTGAA	
CN655/1480	CTC_00194	mfd	P2359-F	CCGCTGCAGGAGGTGAATTTTATTATGAGAT	236
			P2360-R	GGCCATGGAATATTTTTTGCTTCTATATCG	

**Table 3:** Primers used for qRT-PCR, protein expression and promoter regions of *tent* and *tetR* for EMSA experiments.

Target Gene	Primer	Nucleotide Sequence (5′-->3)	Product Length (bp)
**qRT-PCR**			
*tent*	P1714-F	CCAAGGTGCACAAGGAATTT	146
	P1715-R	CAATGTTTAATGCGGGTCCT	
*tetR*	P1726-F	GTTGCTCAAATTATTTAAACTTCGAA	115
	P1727-R	GCTATATCACATTCTTTCATATCTTCAAA	
*rpoB*	P2142-F	TTGAAGAATGTAAAGAGAGAGATGCTAC	118
	P2143-R	GGGAAGTCACCCATAAAGACA	
*gyrA*	P2146-F	AAGATGATGTAGCAGTAAGTATGGA	98
	P2147-R	CTCTGAAGCCAATGTCCTTTT	
**Recombinant protein expression**			
CTC_p21	P2349-F	CGCCGCGGATCCATGTATAAGATATTGATTGTTGAA	711
	P2350-R	CCGCCGGAATTCTTACACCTGAAATAAACGATAGCC	
CTC_01979	P2351-F	CGCCGCGGATCCATGAACAAAATAAATTGTGTAATAATA	792
	P2352-R	CCGCCGGAATTCTTAAAAATCTAATATGTCCTTTAAGTG	
CTC_01421	P2353-F	CGCCGCGGATCCGTGAACAACATATTGTTAGTTGAA	717
	P2354-R	CCGCCGGAATTCCTATTTATTAATTTCGTAGTTCCACCT	
codY	P2355-F	CGCGGATCCATGTCATCATTATTAGAGAAG	801
	P2356-R	CCGCCGGAATTCTTACTTAATTTTTTTCAATTCCTC	
CTC_00935	P2357-F	CGCGGATCCGTGTGTAGAGTAGTGCTT	759
	P2358-R	CCGCCGGAATTCTTATACTTTTTTATTATTCAC	
**EMSA**			
P*tent*	P2365-F	(5’-end labelled biotin) GGTGGCTCCATCATAATAATTGTAT	359
	P2366-R	(5’-end labelled biotin) GGTTTTAGCATTAAAAAAATTAGAACCTA	
P*tetR*	P2363-F	(5’-end labelled biotin) CAGTATTTTTGAAATGTATAATAATTACTTC	316
	P2364-R	(5’-end labelled biotin) CGGTTCTCTTAATTTAGTAATATCAATAT	

F, forward; R, reverse.

## Supplementary Materials

The following are available online at https://www.mdpi.com/2072-6651/12/5/328/s1: Figure S1: Growth kinetics and tetanus toxin (TeNT) production in CN655 wt and CN655/pAT18. Growth kinetics (A) of CN655/pAT18 was slightly lower than that of CN655 wt during the first 56 h, but the total production of TeNT (B) was slightly higher. In the stationary phase from 72 to 144 h, growth kinetics and total TeNT production were similar in both CN655/pAT18 and CN655 wt, Data are mean values ± SEM of at least three independent cultures. Figure S2: Extracellular tetanus toxin (TeNT) produced by CN655/pAT18 (empty vector) and CN655 anti-sense strains showing no significant difference. CN655/p1308, CN655/p1309, CN655/p1312, CN655/p1313, CN655/p1472 and CN655/p1480 showed no significant difference in extracellular levels of TeNT compared to CN655/pAT18. Data are mean values ± SEM of at least three independent cultures. Figure S3: Total tetanus toxin (TeNT) produced by CN655/pAT18 (empty vector) and CN655 anti-sense strains showing no significant difference. CN655/1308, CN655/p1309, CN655/p1310, CN655/p1311, CN655/p1312 and CN655/p1313 showed no significant difference in total TeNT levels compared to CN655/pAT18. Data are mean values ± SEM of at least three independent cultures. Figure S4: Expression of (A) *tent* and (B) *tetR* in CN655/pAT18 and CN655 anti-sense strains showing no significant difference. (A) CN655/p1308, CN655/p1309, CN655/p1312 and CN655/p1313 showed no significant difference in *tent* expression compared to CN655/pAT19. (B) CN655/p1308 and CN655/p1309 showed no significant difference in *tetR* expression compared to CN655/pAT19. Data are mean values ± SEM of at least three independent cultures. Figure S5: Growth kinetics and tetanus toxin (TeNT) production in CN655 wt in TGY versus TY culture medium. The presence of glucose (5 g/L) in culture medium (TGY) induced a slightly increased growth and a more than two-fold extracellular TeNT compared to TY medium without supplementation in glucose. Data are mean values ± SEM of at least three independent cultures. Figure S6: Extracellular tetanus toxin (TeNT) produced by CN655/p1311 and CN655/p1313 grown in TGY supplemented with inorganic phosphate. CN655/p1311 and CN655/p131 were grown in TGY and in TGY supplemented with 40 mM Na_2_HPO_4_. Extracellular TeNT was monitored by ELISA. Data are mean values ± SEM of at least three independent assays.
