# Supplementary material for: Tetanus Toxin Synthesis is Under the Control of A Complex Network of Regulatory Genes in Clostridium tetani
Source: Toxins (Basel). 2020 May 15;12(5):328. doi: 10.3390/toxins12050328 (PMC7290440; doi:10.3390/toxins12050328)
Supplement: Supplementary file 1 [file toxins-12-00328-s001.pdf]

Supplementary Materials: Tetanus Toxin Synthesis is Under the Control of A Complex Network of Regulatory Genes in *Clostridium tetani*

Diana Chapeton-Montes, Lucile Plourde, Cecile Deneve, Dominique Garnier, Fabien Barbirato, Vincent Colombié, Sandy Demay, Georges Haustant, Olivier Gorgette, Christine Schmitt, Catherine Thouvenot, Holger Brüggemann and Michel R. Popoff

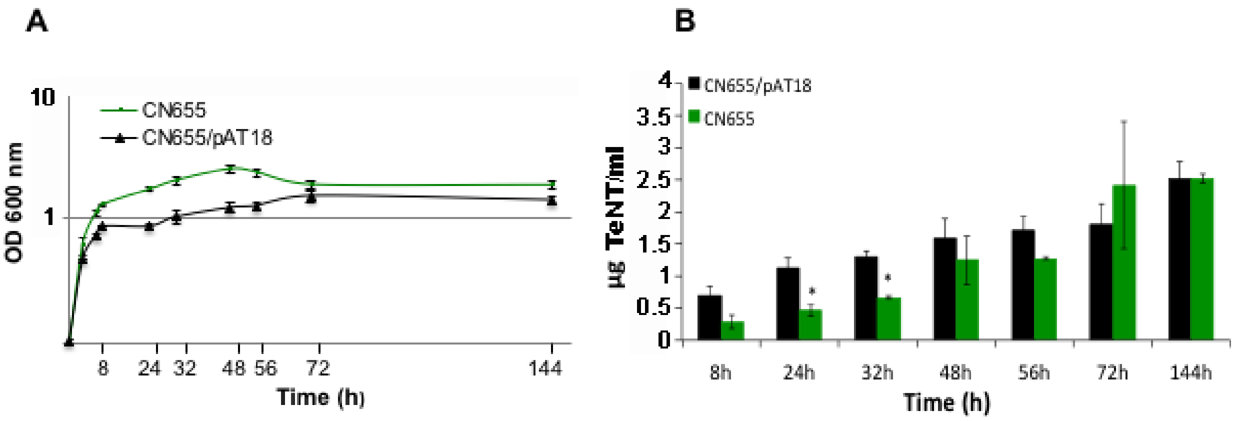

Figure S1. Growth kinetics and tetanus toxin (TeNT) production in CN655 wt and CN655/pAT18. Growth kinetics (A) of CN655/pAT18 was slightly lower than that of CN655 wt during the first 56 h, but the total production of TeNT (B) was slightly higher. In the stationary phase from 72 to 144 h, growth kinetics and total TeNT production were similar in both CN655/pAT18 and CN655 wt, Data are mean values ± SEM of at least three independent cultures.

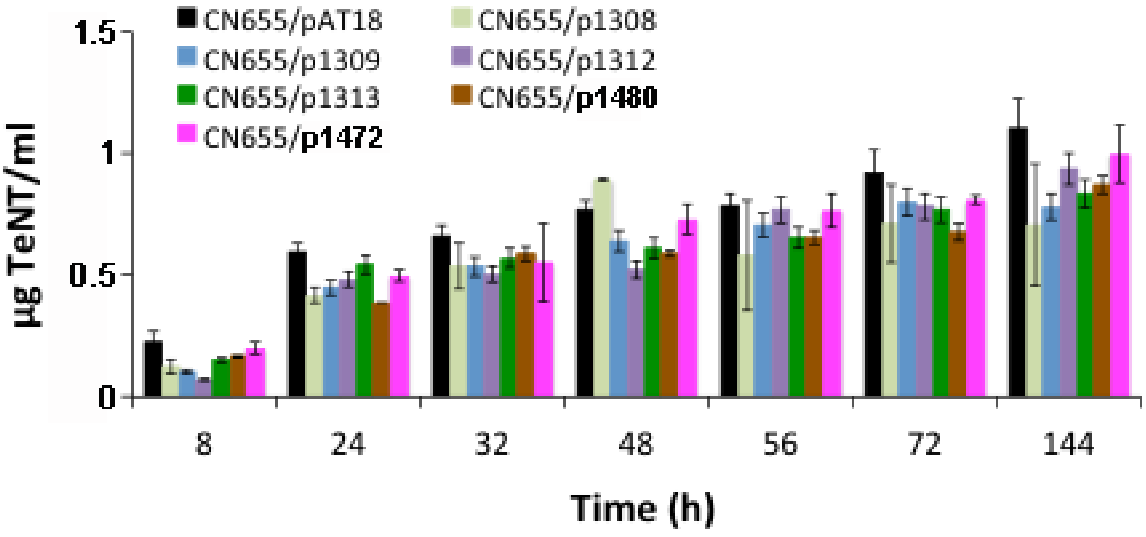

Figure S2. Extracellular tetanus toxin (TeNT) produced by CN655/pAT18 (empty vector) and CN655 antisense strains showing no significant difference. CN655/p1308, CN655/p1309, CN655/p1312, CN655/p1313, CN655/p1472, CN655/p1480 showed no significant difference in extracellular levels of TeNT compared to CN655/pAT18. Data are mean values ± SEM of at least three independent cultures,.

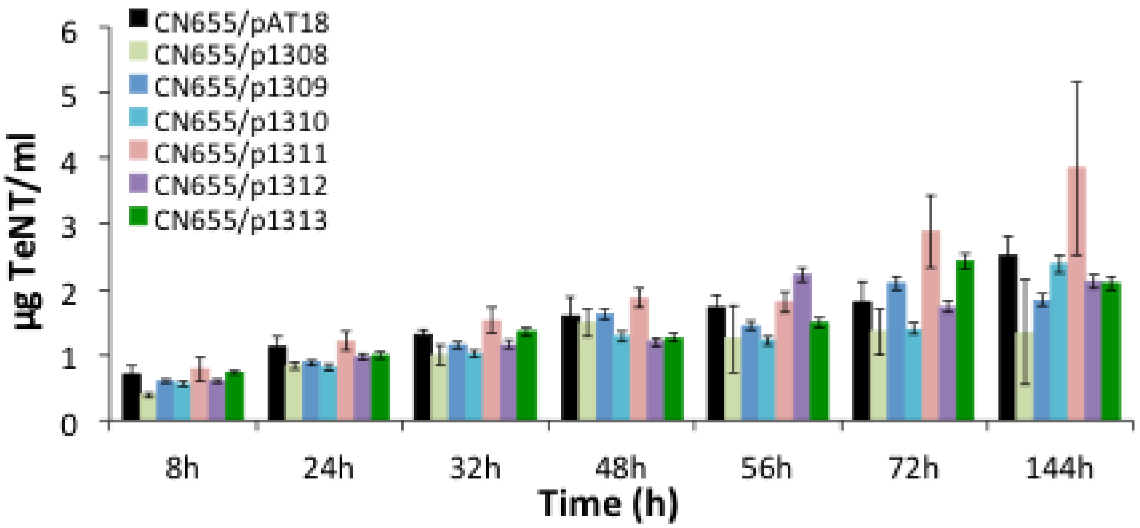

Figure S3. Total tetanus toxin (TeNT) produced by CN655/pAT18 (empty vector) and CN655 antisense strains showing no significant difference. CN655/1308, CN655/p1309, CN655/p1310, CN655/p1311, CN655/p1312, CN655/p1313 showed no significant difference in total TeNT levels compared to CN655/pAT18. Data are mean values ± SEM of at least three independent cultures,.

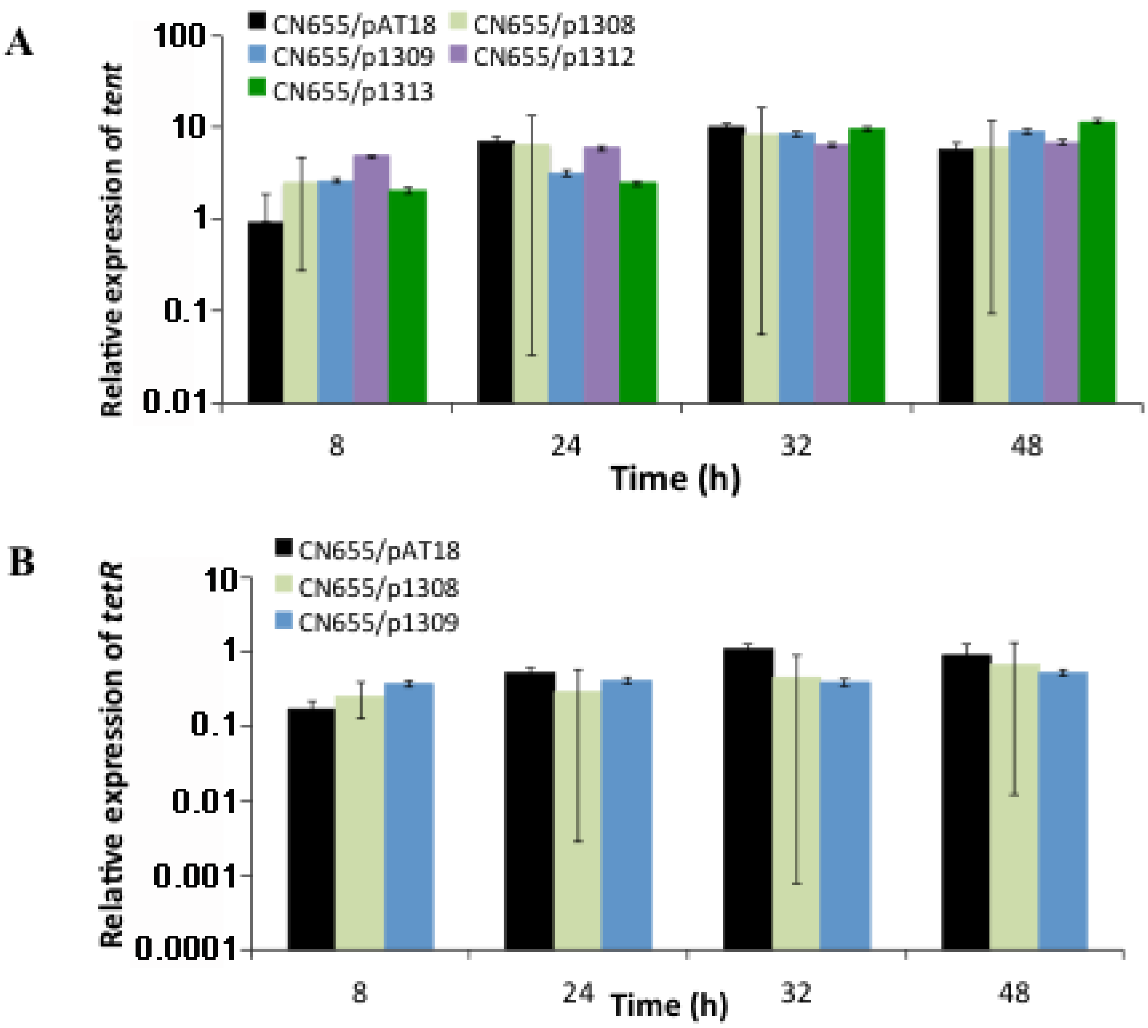

Figure S4. Expression of (A) *tent* and (B) *tetR* in CN655/pAT18 and CN655 antisense strains showing no significant difference. (A) CN655/p1308, CN655/p1309, CN655/p1312, and CN655/p1313 showed no significant difference in *tent* expression compared to CN655/pAT19. (B) CN655/p1308 and CN655/p1309 showed no significant difference in *tetR* expression compared to CN655/pAT19. Data are mean values ± SEM of at least three independent cultures,.

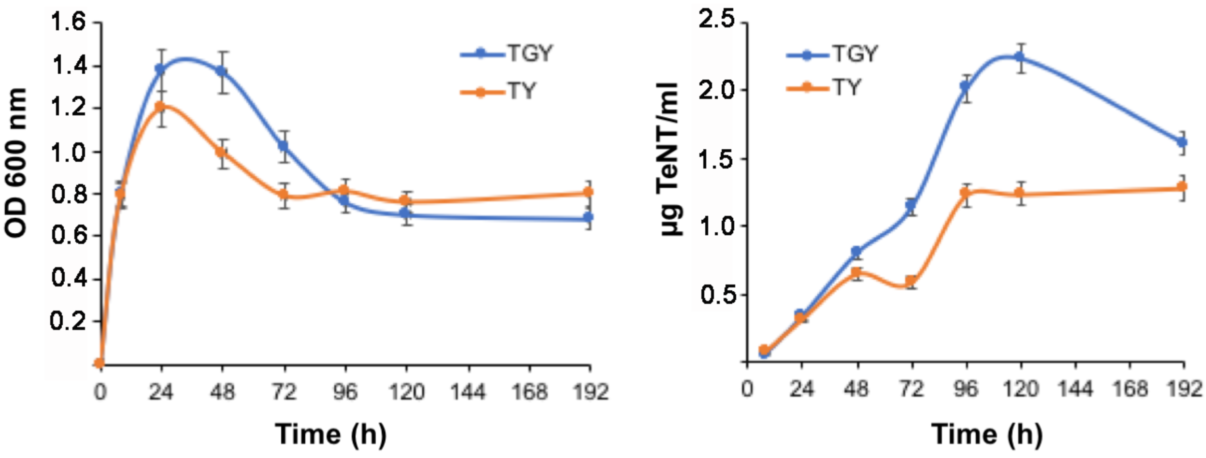

Figure S5. Growth kinetics and tetanus toxin (TeNT) production in CN655 wt in TGY versus TY culture medium. The presence of glucose (5 g/L) in culture medium (TGY) induced a slight increased growth and a more than two-fold extracellular TeNT compared to TY medium without supplementation in glucose. Data are mean values ± SEM of at least three independent cultures,.

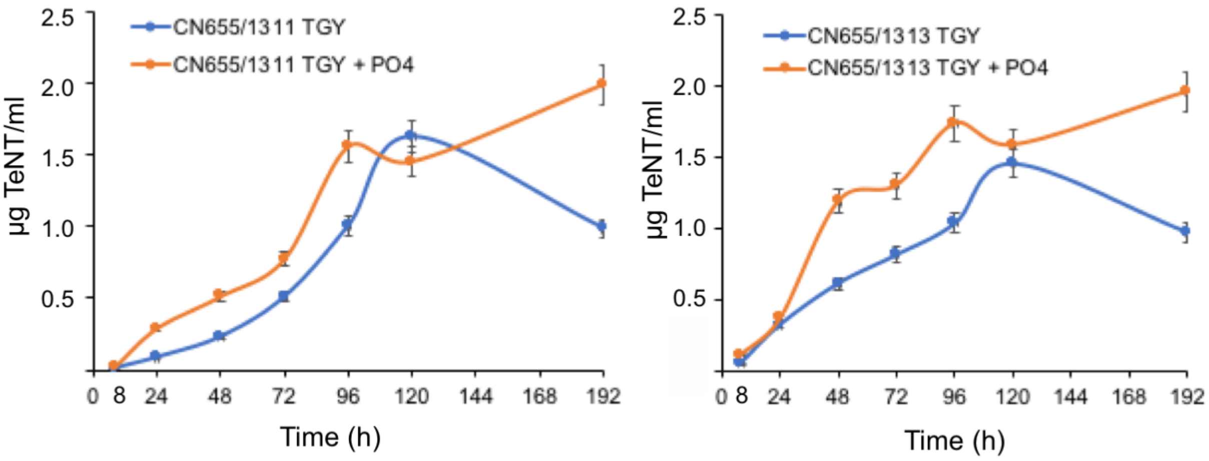

Figure S6. Extracellular tetanus toxin (TeNT) produced by CN655/p1311 and CN655/p1313 grown in TGY supplemented with inorganic phosphate. CN655/p1311 and CN655/p131 were grown in TGY and in TGY supplemented with 40 mM Na_2HPO_4. Extracellular TeNT was monitored by ELISA. Data are mean values ± SEM of at least three independent assays.
